# Phylogenetic relationships in the *Sorghum* genus based on sequencing of the chloroplast and nuclear genes

**DOI:** 10.1002/tpg2.20123

**Published:** 2021-07-29

**Authors:** Galaihalage Ananda, Sally Norton, Cecilia Blomstedt, Agnelo Furtado, Birger Møller, Roslyn Gleadow, Robert Henry

**Affiliations:** ^1^ Queensland Alliance for Agriculture and Food Innovation The Univ. of Queensland St Lucia QLD Australia; ^2^ Australian Grains Genebank Agriculture Victoria Horsham VIC Australia; ^3^ School of Biological Sciences Monash Univ. Clayton VIC Australia; ^4^ Plant Biochemistry Laboratory, Dep. of Plant and Environmental Sciences Univ. of Copenhagen Copenhagen Denmark

## Abstract

Sorghum [*Sorghum bicolor* (L.) Moench] is an important food crop with a diverse gene pool residing in its wild relatives. A total of 15 sorghum accessions from the unexploited wild gene pool of the *Sorghum* genus, representing the five subgenera, were sequenced, and the complete chloroplast genomes and 99 common single‐copy concatenated nuclear genes were assembled. Annotation of the chloroplast genomes identified a total of 81 protein‐coding genes, 38 tRNA, and four rRNA genes. The gene content and gene order among the species was identical. A total of 153 nonsynonymous amino acid changes in 40 genes were identified across the species. Phylogenetic analysis of both the whole chloroplast genome and nuclear genes revealed a similar topology with two distinct clades within the genus. The species within the subgenera *Eusorghum*, *Chaetosorghum*, and *Heterosorghum* clustered in one clade, whereas the species within the subgenera *Parasorghum* and *Stiposorghum* clustered in a second clade. However, the subgenera *Parasorghum* and *Stiposorghum* were not monophyletic, suggesting the need for further research to resolve the relationships within this group. The close relationship between the two monotypic subgenera *Chaetosorghum* and *Heterosorghum* suggests that species within these subgenera could be considered as one group. This analysis provides an improved understanding of the genetic relationships within the *Sorghum* genus and defines diversity in wild sorghum species that may be useful for crop improvement.

## Core Ideas

- Wild and domesticated sorghum chloroplast genomes have the same gene content and order.
- Chloroplast and nuclear phylogenies resulted in similar trees.
- Chloroplast and nuclear phylogenies resolved the *Sorghum* genus into two distinct clades.

**Abbreviations**
CDS: coding DNA sequence
CLC‐GWB: CLC Genomic Workbench
CpGAVAS: Chloroplast Genome Annotation, Visualization, Analysis, and GenBank Submission
IR: inverted repeats
ML: maximum likelihood
NCBI: National Center for Biotechnology Information
SNP: single nucleotide polymorphism

## 1 INTRODUCTION

Sorghum [*Sorghum bicolor* (L.) Moench] is a versatile food crop globally and a staple food in many African and Asian countries, whereas elsewhere it is mainly grown for animal feed (Hariprasanna & Patil, 2015; Venkateswaran et al., 2019). *Sorghum* (Poaceae) is a highly diverse genus reported to consist of 24 species (Ananda et al., 2020), that, based on morphological characteristics are classified into five subgenera: *Eusorghum*, *Chaetosorghum*, *Heterosorghum*, *Parasorghum*, and *Stiposorghum* (de Wet, 1978; Garber & Snyder, 1950; Harlan & de Wet, 1972; Lazarides et al., 1991). In the current Kew Botanic Gardens Angiosperm DNA C‐values database, a total of 32 species of sorghum are listed (https://cvalues.science.kew.org/search/angiosperm), based on several studies. Thus, the exact number of species is still uncertain. Of the wild sorghum species, 17 are native to Australia (Myrans et al., 2020). Although the genome of domesticated sorghum has been well characterized (Paterson et al., 2009), limited genomic data are available on this diverse gene pool of Australian wild sorghum species.

The classification of sorghum based on diverse morphological characters has made sorghum taxonomy a complex area of study. In the present classification based on cytogenetics and morphology, *Eusorghum* contains domesticated sorghum, the cultivated species *S. bicolor*, along with its progenitors and weedy relatives *S. propinquum* (Kunth) Hitchc. and *S. halepense* (L.) Pers. (de Wet, 1978; Harlan & de Wet, 1972). *Sorghum *×* almum* Parodi is a hybrid species in *Eusorghum* (Dillon et al., 2007a) and thought to be a hybrid between *S. bicolor* and *S. halepense* (Duvall & Doebley, 1990). *Sorghum bicolor* includes all the cultivated sorghum species, which is subdivided into three categories; subsp. *bicolor* (cultivated sorghums), subsp. *verticilliflorum* (wild progenitors of sorghum), and subsp. *drummondii* (Steud.) Millsp. & Chase (weedy sorghum hybrids and the species derived from hybridization between domesticated sorghums and wild relatives). Subspecies *verticilliflorum* consists of four races of wild progenitors named *aethiopicum*, *arundinaceum*, *verticilliflorum*, and *virgatum*. *Sorghum propinquum* is a wild species, whereas *S. halepense* is a weedy species. The subgenus *Parasorghum* consists of seven species: *S. grande* Lazarides, *S. nitidum* (Vahl) Pers., *S. matarankense* E. D. Garber & Snyder, *S. leiocladum* (Hack.) C. E. Hubb., *S. purpureosericeum* (Hochst. ex A. Rich.), *S. timorense* (Kunth) Buse, and *S. versicolor* Andersson*. Sorghum amplum* Lazarides, *S. brachypodum* Lazarides, *S. angustum* S. T. Blake, *S. intrans* F. Muell. ex Benth., *S. ecarinatum* Lazarides, *S. bulbosum* Lazarides, *S. plumosum* (R. Br.) P. Beauv., *S. stipoideum* (Ewart & Jean White) C. A. Gardner & C. E. Hubb., *S. interjectum* Lazarides, and *S. exstans* Lazarides are classified under the subgenus *Stiposorghum*. *Sorghum laxiflorum* F. M. Bailey, and *S. macrospermum* E. D. Garber belong to monotypic subgenera *Heterosorghum* and *Chaetosorghum*, respectively (Ananda et al., 2020; Lazarides et al., 1991; USDA‐ARS, National Plant Germplasm System, 2020). The majority of the species in the subgenera of *Chaetosorghum*, *Heterosorghum*, *Parasorghum*, and *Stiposorghum* are endemic to Australia.

Molecular phylogenetics provides new insights into the evolutionary relationships within species. There have been several studies aimed at determining the phylogenetic relationships in sorghum (Dillon et al., 2001, 2004; Liu et al., 2014; Sun et al., 1994, 2007a). However, understanding the precise relationships of these subgenera has been challenging. The main reasons for the uncertainty around the conclusions are the limited availability of sequencing data and the use of different sets of taxa and different sets of DNA markers in the studies (Kellogg, 2013). To date, there are some studies on the phylogeny of sorghum based on nuclear genomic information together with some chloroplast genomic data (Dillon et al., 2001, 2004; Liu et al., 2014; Sun et al., 1994, 2007a). No information is available based on whole chloroplast genome sequencing or multiple nuclear genes. In an attempt to reconcile the different proposed phylogenies, Spangler (2003) revised the *Sorghum* genus, dividing it into three distinct genera, namely, *Sorghum*, *Vacoparis*, and *Sarga*. However, this revised classification has been questioned (Dillon et al., 2004, 2007a) and the precise relationships among these species remain unclear.

**Core Ideas**

- Wild and domesticated sorghum chloroplast genomes have the same gene content and order.
- Chloroplast and nuclear phylogenies resulted in similar trees.
- Chloroplast and nuclear phylogenies resolved the *Sorghum* genus into two distinct clades.

Resolving the complex relationships in the genus *Sorghum* requires accurate genetic evidence. Advances in DNA sequencing have enabled large volumes of reliable genomic data to be obtained (Bennetzen et al., 2012). The first whole genome sequence of sorghum was obtained from the grain sorghum, *S. bicolor* BTx623, using whole‐genome shotgun sequencing (Paterson et al., 2009). The total size of the genome is 730 Mb, and this small size makes it a perfect candidate as a model genome for C4 grasses. The chloroplast genomes of plants have been successfully used in revealing phylogenetic relationships of plants because of their highly conserved nature (Dong et al., 2017; Jansen et al., 2005). The first physical map of a chloroplast genome was generated for maize (*Zea mays* L.) (Sugiura, 2003). Some portions of the chloroplast genomes are found present in the genomes of other organelles, such as the mitochondria and nucleus (Sugiura, 1992). The typical chloroplast genome is a single circular structure including a large single‐copy region and a small single‐copy region. Those two regions are separated by two inverted repeats (IRa and IRb) (Jansen et al., 2005). A chloroplast genome can contain about 120–135 genes, of which the majority (65%) are protein coding genes with the rest being RNA encoding genes (Lopez‐Juez & Pyke, 2005). Plant cells may contain 10,000 copies of the chloroplast genome (Morley & Nielsen, 2016). However, the gene order and the coding sequences are highly conserved within species (Jansen et al., 2005; Palmer, 1991). The first successful attempt at sequencing the chloroplast genome of sorghum was by Saski et al. (2007). To date, the complete chloroplast genomes of only seven sorghum species of just two subgenera, *Eusorghum* and *Parasorghum*, are available in databases, leaving the gene pool of the Australian wild sorghum species unexploited. In this study, we determine phylogenetic relationships in the genus *Sorghum* using whole genome sequencing to capture the entire chloroplast genome sequence and a large set of nuclear gene sequence data of Australian wild sorghum species.

## 2 MATERIALS AND METHODS

### 2.1 Plant material

A total of 15 sorghum accessions of seven sorghum species representing five subgenera were selected for sequencing (Table 1). Plants were grown at the Australian Grains Genebank, Horsham, VIC, Australia (36°43′21.93764” S and 142°10′29.50331″ E). Seed was germinated according to the Australian Grains Genebank protocol, modified by the addition of a 16‐h incubation in 3 mM gibberellic acid (Cowan et al., 2020). Seedlings were transferred to commercial seed raising mix and grown for 4 wk in a growth cabinet with alternating temperatures of 35 °C/25 °C for 16/8 h and a 14‐h photoperiod. The plants were then transplanted into 25‐cm pots containing commercial potting mix and grown for a further 4 wk in the growth cabinet and then moved into a greenhouse with a maximum temperature of approximately 30 °C and a minimum temperature of approximately 15 °C and a natural photoperiod of 14 h of light.

**TABLE 1:** *Sorghum* genotypes used for sequencing, sourced from the Australian Grains Genebank (AGG)

**AGG** * **accession number**	**Accession name**	**Taxon**	**Subgenera**	**Ploidy level (2*n*)**	**Country of origin**	**NCBI BioSample number**
302367	JC 2253	*S. macrospermum*	*Chaetosorghum*	40	Australia	SAMN17369313
326072	SSD line derived from AGG 302367 WSOR	*S. macrospermum*	*Chaetosorghum*	40	Australia	SAMN17369304
112151	Tx623 B	*S. bicolor*	*Eusorghum*	20	Africa	SAMN17369308
314746	Tx623 A	*S. bicolor*	*Eusorghum*	20	Africa	SAMN17369307
326060	SSD line derived from AGG 302525 WSOR	*S. laxiflorum*	*Heterosorghum*	40	Australia	SAMN17369311
326074	SSD line derived from AGG 302525 WSOR	*S. laxiflorum*	*Heterosorghum*	40	Australia	SAMN17369317
326061	SSD line derived from AGG 300148 WSOR	*S. leiocladum*	*Parasorghum*	10,20	Australia	SAMN17369303
326062	SSD line derived from AGG 300148 WSOR	*S. leiocladum*	*Parasorghum*	10,20	Australia	SAMN17369312
326065	SSD line derived from AGG 302521 WSOR	*S. matarankense*	*Parasorghum*	10	Australia	SAMN17369305
326066	SSD line derived from AGG 302521 WSOR	*S. matarankense*	*Parasorghum*	10	Australia	SAMN17369314
326068	SSD line derived from AGG 318068 WSOR	*S. purpureosericeum*	*Parasorghum*	10	Africa	SAMN17369306
326071	SSD line derived from AGG 318068 WSOR	*S. purpureosericeum*	*Parasorghum*	10	Africa	SAMN17369315
326075	SSD line derived from AGG 318068 WSOR	*S. purpureosericeum*	*Parasorghum*	10	Africa	SAMN17369316
302670	IDC 7561	*S. brachypodum*	*Stiposorghum*	10	Australia	SAMN17369310
326073	SSD line derived from AGG 302481 WSOR	*S. brachypodum*	*Stiposorghum*	10	Australia	SAMN17369309

*Note*. NCBI, National Center for Biotechnology Information; SSD = single seed descent.

### 2.2 DNA extraction and sequencing

Total genomic DNA was extracted from pulverized leaf tissue samples of the 15 sorghum accessions using the cetyltrimethyl ammonium bromide method optimized for sorghum (Furtado, 2014). DNA quality was assessed by spectrometry using A260/280 and A260/230 absorbance ratios (NanoDrop, Thermofisher Scientific) and by resolving the DNA on an agarose gel by electrophoresis. DNA quantity was measured by SYBR‐safe fluorescence comparison to lambda DNA standards. A ratio of 1.8:2 in the absorbance at 260/280 nm in the Nano drop profiles and no shearing indicated high molecular weight DNA with sufficient purity and quality of DNA required for Illumina sequencing. DNA samples were sequenced on an Illumina *HiSeq* 2000 platform at the Ramaciotti Centre, University of New South Wales, NSW, Australia (Supplemental Table S1).

### 2.3 Chloroplast genome assembly

Chloroplast genomes of each species were assembled using an establised analysis pipeline described in Moner et al. (2018) in CLC Genomic Workbench (CLC‐GWB) software (CLC Genomics Workbench 11.0, http://www.clcbio.com). Raw reads were imported to CLC‐GWB, and the sequence of *S. bicolor* chloroplast genome (accession *NC008602*) was imported from the National Center for Biotechnology Information (NCBI) to use as the reference (Saski et al., 2007). Paired reads were quality trimmed at 0.01 quality limits (Phred score equivalent to >20, 95% of the reads had a Phred score >35, averaged across all bases) at sequence length of 1,000 to assemble the chloroplast genome by two approaches, namely reference‐guided mapping assembly and de novo assembly. A manual curation step was carried out by checking the mapping files to reconcile any discrepancies, thereby obtaining an accurate sequence, ensuring a high‐quality whole chloroplast genome sequence for all the species.

### 2.4 Genome annotation and variant analysis

The chloroplast genomes were annotated using the GeSeq online tool (https://chlorobox.mpimp‐golm.mpg.de/geseq.html) using *S. bicolor* as the reference genome. The variants compared with the reference were identified using Geneious 11.1.5 software (www.geneious.com/). The annotated sequences were exported to Geneious 11.1.5 and each species was separately aligned against the annotated reference genome of *S. bicolor* to identify the variants.

### 2.5 Nuclear genes analysis

The nuclear gene sequences of the 15 sorghum accessions were constructed by an established analysis pipeline described in Badro et al. (2019) in CLC‐GWB software. Raw reads were imported to CLC‐GWB together with the annotated nuclear genome sequence of *S. bicolor NCBIv3* (Paterson et al., 2009) and the *Zea mays* genomes to use as a reference and an outgroup, respectively. First, raw reads were subjected to quality control analysis, and trimming was performed at the quality limits of 0.01 (Phred score equivalent to >20) at sequence length of 1,000 and the reads were mapped against the *S. bicolor* reference genome. The consensus sequences were extracted for each species and converted into coding DNA sequence (CDS) and genome tracks. From the CDS and genome tracks, annotations were extracted, and a total of 99 common single‐copy genes were selected (Li et al., 2017) (Supplemental Table S2). These genes (exons only) were concatenated to give a final annotated sequence per species.

### 2.6 Phylogenetic analysis

Phylogenetic analysis was undertaken separately using whole chloroplast genome sequence datasets and nuclear sequence datasets of the sorghum species. Geneious 11.1.5 software (www.geneious.com) was used to align the sequences. In the chloroplast genome alignment, the complete chloroplast sequences of an additional seven species were imported from NCBI (*S. arundinaceum LS398103*, *S. halepense LS398105*, *S. propinquum NC042789*, *S*. × *drummondii LS398106, S. sudanense NC042790, S. versicolor LS398104*, and *S. timorense LS398107*) and the multiple sequence alignment was done using the multiple alignment using fast Fourier transform (MAFFT) alignment in Geneious with default parameters. The substitution models for the phylogenetic analyses were determined by the jModelTest2 (Darriba et al., 2012) on the Extreme Science and Engineering Discovery Environment (XSEDE). The parameters for the best selected models are given in the Supplemental Tables S3 and S4. Phylogenetic reconstruction was performed using PAUP* v. 4.0 software (Swofford, 2003) with maximum likelihood (ML) and maximum parsimony methods. The ML tree was constructed using the best fit model under the Akaike information criterion (Supplemental Tables S3 and S4) in PAUP* with heuristic searching, tree bisection and reconnection branch swapping method, and 1,000 bootstrap replicates. The final trees obtained were cross‐validated for the similar tree topology using MrBayes v. 3.2 (Ronquist et al., 2012) in Geneious with the parameters of Bayesian information criterion in jModelTest2 (Supplemental Tables S3 and S4). The nuclear and chloroplast phylogenies were compared to identify differences.

## 3 RESULTS

### 3.1 Chloroplast genome assembly and annotation

**FIGURE 1:**
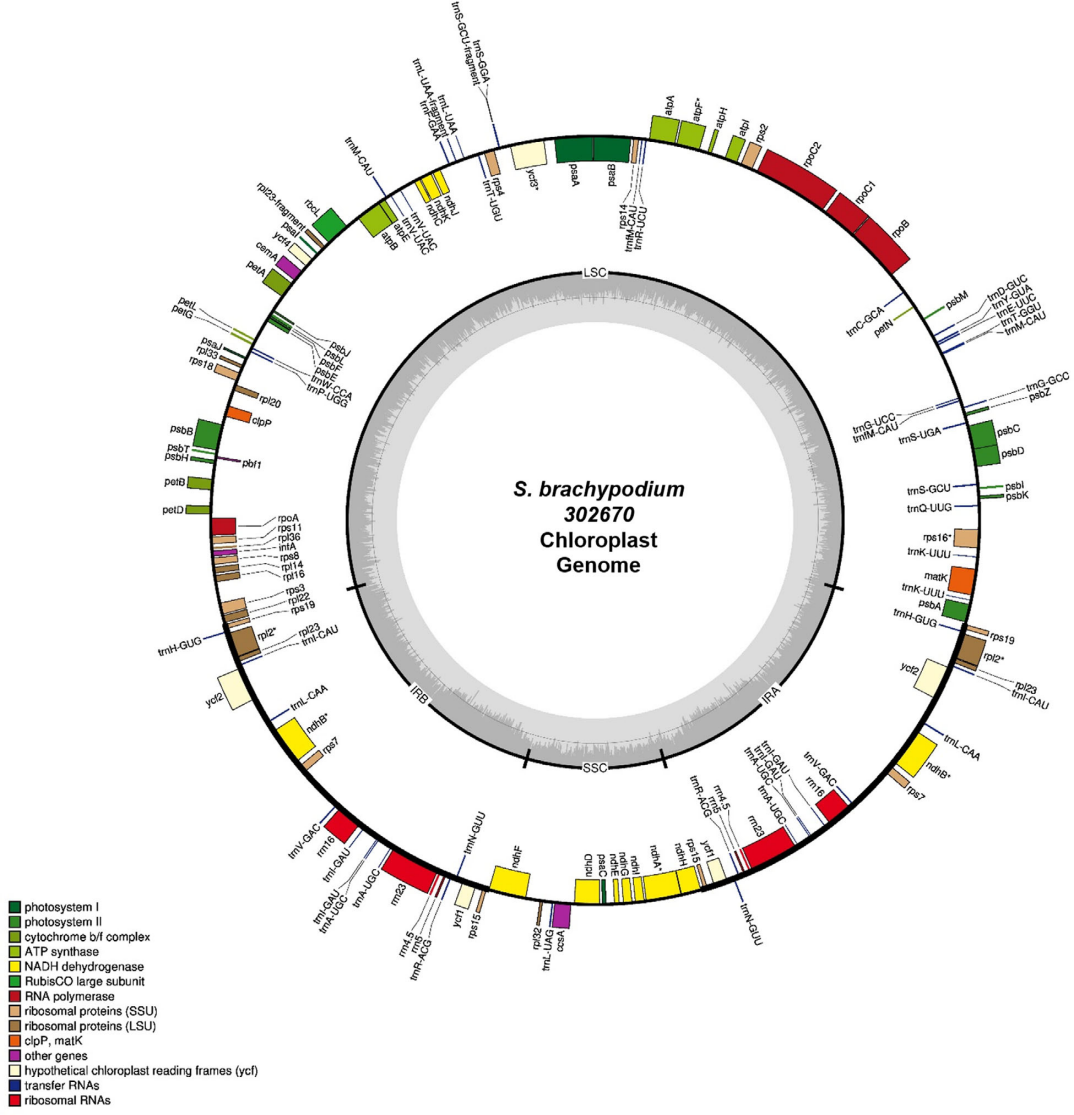
Chloroplast genome map of *Sorghum brachypodum* 3*02670*. Chloroplast genome map drawn using the GeSeq software. The genes belonging to different functional groups are indicated in different colors. The thicker lines indicate the extent of the inverted repeats (IR). Genes inside the circle are transcribed in the clockwise direction whereas the genes outside the circle are transcribed in the counter clockwise direction. LSC, large single copy, SSC, small single copy

Illumina sequencing generated a total of 68,805,710–537,816,756 raw reads from each of the different species with a mean length of 150 bp. The paired end reads trimmed at 0.01 quality limit (Phred score >20), with reads ranging from 66,638,305 to 516,800,277 at an average coverage of 20× of the genome size of the corresponding species, were selected for assembling the chloroplast genome sequences (Supplemental Table S1). The chloroplast genomes were assembled separately for each species and ranged in size from 140,666 to 141,010 bp (Table 2). The *S. bicolor* chloroplast genome sequence *NC008602* was used as a reference sequence for comparison to the assembled and annotated chloroplast genome sequences. This revealed that the structure of the chloroplast genome sequences of all the sorghum species were identical, with a quadripartite structure including a large single‐copy (82,596–82,922 bp), small single‐copy (12,486–12,565 bp), and two identical IRs (22,782–22,813 bp). The overall guanine or cytosine content of the seven species were almost the same (38.4–38.5%). A well‐conserved genomic structure was observed among the seven species with identical content and order of genes (Table 2). The chloroplast genome map of *S. brachypodum 302670* is presented in Figure 1, which represents the structure of chloroplast genome of sorghum species.

**TABLE 2:** Annotated complete chloroplast genomes of the sorghum species

						**Total number of**			
**Taxon**	**Size of the genome**	**Overall GC content**	**LSC**	**SSC**	**IR**	**Genes**	**CDSs**	**rRNA genes**	**tRNA genes**
	bp		bp						
*S. macrospermum 302367*	140,997	38.4	82,842	12,565	22,795	123	81	4	38
*S. macrospermum 326072*	140,983	38.4	82,854	12,529	22,800	123	81	4	38
*S. bicolor 112151*	140,754	38.5	82,685	12,503	22,783	123	81	4	38
*S. bicolor 314746*	140,666	38.5	82,596	12,506	22,782	123	81	4	38
*S. laxiflorum 326060*	140,992	38.4	82,853	12,537	22,801	123	81	4	38
*S. laxiflorum 326074*	140,992	38.4	82,853	12,537	22,801	123	81	4	38
*S. leiocladum 326061*	140,714	38.5	82,630	12,486	22,799	123	81	4	38
*S. leiocladum 326062*	140,820	38.5	82,736	12,486	22,799	123	81	4	38
*S. matarankense 326065*	140,710	38.5	82,605	12,489	22,808	123	81	4	38
*S. matarankense 326066*	140,710	38.5	82,605	12,489	22,808	123	81	4	38
*S. purpureosericeum* *326068*	141,010	38.4	82,922	12,488	22,800	123	81	4	38
*S. purpureosericeum* *326071*	140,928	38.5	82,840	12,488	22,800	123	81	4	38
*S. purpureosericeum* *326075*	140,989	38.5	82,901	12,488	22,800	123	81	4	38
*S. brachypodum* *302670*	140,701	38.5	82,599	12,486	22,808	123	81	4	38
*S. brachypodum* *326073*	140,734	38.5	82,621	12,487	22,813	123	81	4	38

*Note*. CDSs, coding DNA sequences; GC, guanine or cytosine; IR, inverted repeats; LSC, large single copy; SSC, small single copy.

**FIGURE 2:**
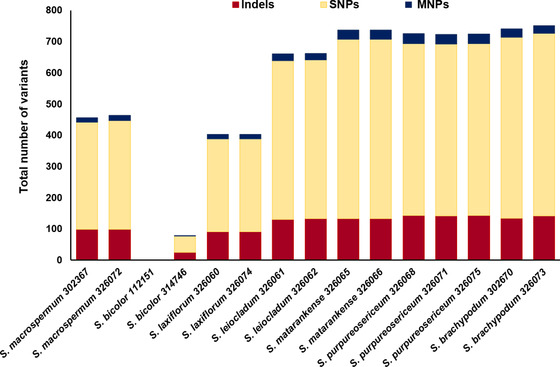
Indels, single nucleotide polymorphism (SNPs), and multiple nucleotide polymorphisms (MNPs) inside and outside CDS per species. The total number of variants including the number of Indels, SNPs, and MNPs per species are given. Variants were determined compared with the reference chloroplast genome sequence of *Sorghum bicolor NC008602*

A total of 123 genes were identified in the sorghum chloroplast genome, including 81 protein coding genes, 38 tRNA genes, and 4 rRNA genes. The annotated chloroplast genomes were imported to Geneious to identify variants compared with the reference genome, *S. bicolor NC008602*. The highest number of total variants was observed in *S. brachypodum 326073* with no variants observed in *S. bicolor 112151*. Among these variants, 75% were single nucleotide polymorphisms (SNPs) and 21% of the variants were Indels across all the species. Similarly, in terms of variants inside coding sequences, the highest number of total variants was observed in the same *S. brachypodum 326073* with no variants observed in *S. bicolor 112151*. Among these variants, 96% of them were SNPs followed by 2% of Indels across all the species. The highest number of nonsynonymous amino acid changes were observed in *S*. *brachypodum 326073* with none observed in *S. bicolor 112151* (Table 3, Figure 2).

**TABLE 3:** Details of sequence polymorphisms identified in chloroplast genome sequences of sorghum species

	**Number of variants inside and outside CDS**				**Number of variants inside CDS**				
**Species**	**Total variants**	**Indels**	**SNPs**	**MNPs**	**Total variants**	**Indels**	**SNPs**	**MNPs**	**Nonsynonymous amino acid changes**
*S. macrospermum 302367*	456	99	343	14	124	2	119	3	37
*S. macrospermum 326072*	464	99	348	17	123	2	116	5	36
*S. bicolor 112151*	0	0	0	0	0	0	0	0	0
*S. bicolor 314746*	78	25	52	1	22	1	20	1	9
*S. laxiflorum 326060*	402	91	298	13	108	1	104	3	31
*S. laxiflorum 326074*	402	91	298	13	108	1	104	3	31
*S. leiocladum 326061*	660	131	508	21	180	2	176	2	58
*S. leiocladum 326062*	662	133	508	21	180	2	176	2	58
*S. matarankense 326065*	736	133	574	29	203	3	197	2	67
*S. matarankense 326066*	736	133	574	29	203	3	197	2	67
*S. purpureosericeum 326068*	725	144	550	31	194	4	186	4	59
*S. purpureosericeum 326071*	723	142	550	31	194	4	186	4	59
*S. purpureosericeum 326075*	724	143	550	31	194	4	186	4	59
*S. brachypodum 302670*	740	135	579	26	206	4	200	2	67
*S. brachypodum 326073*	750	142	584	24	223	4	216	3	78

*Note*. CDS, coding DNA sequence, MNPs, multinucleotide polymorphisms, SNPs, single nucleotide polymorphisms.

**FIGURE 3:**
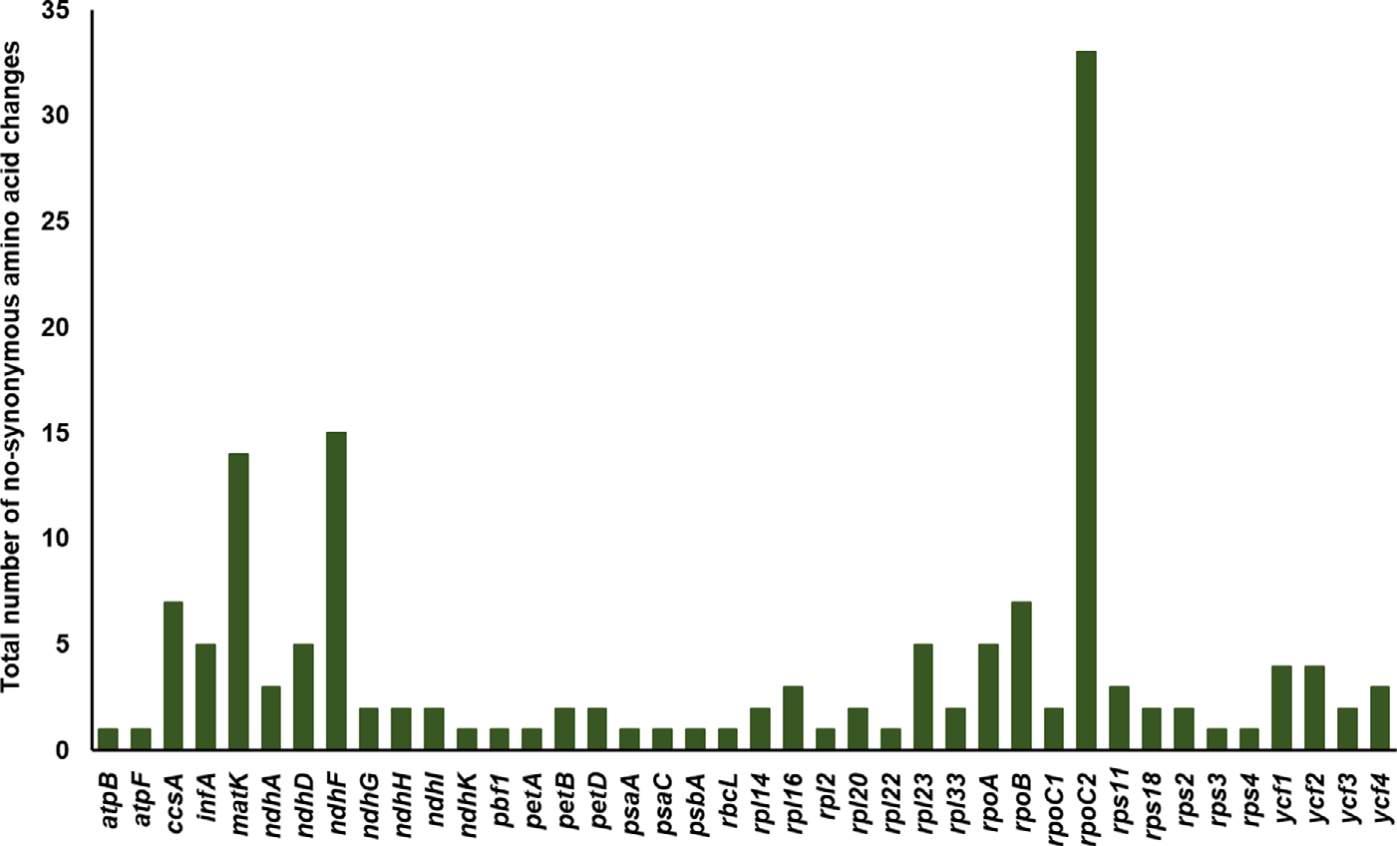
Number of nonsynonymous amino acid changes in chloroplast‐specific genes. The total number of nonsynonymous amino acid changes per gene were determined compared with the reference chloroplast genome sequence of *Sorghum bicolor NC008602*

A total of 153 nonsynonymous amino acid changes were identified in a total of 40 genes among all the 15 accessions while 83 genes showed no variation. The highest number of variants was observed in the gene *rpoC2*, followed by *ndhF* and *matK* (Figure 3). Most of them were present in the coding region of the *rpoC2* gene, which is a DNA‐directed RNA polymerase subunit beta‐coding gene. Two common SNPs were observed in all the species except in *S. bicolor 112151*, a transition of A to G in the gene *matK* and transversion of C to G in gene *ycf4*, which resulted in the amino acid changes Leucine to Serine and Leucine to Valine, respectively. With regard to Indels causing nonsynonymous amino acid changes, in general, the Indels were shared by several species, but there were also some species‐specific Indels. These included a deletion in *rpoC2* in *S. bicolor 314746*, deletions in *rps18* and *rpl22* genes of *S. macrospermum*, and insertions in *ndhD* and *rps3* genes in *S. purpureosericeum* (Table 4). Out of these Indels, 30% caused a frame shift in the final protein of the genes *rpl22*, *rps11*, and *ycf2*, whereas another 30% caused a truncation in the final protein of the genes *matK*, *rps3*, and *ndhD* (Table 4).

**TABLE 4:** Indels present in the coding DNA sequence (CDS) regions of chloroplast genomes of the selected sorghum species

**Species**	**No: of indels**	**Polymorphism type**	**Size**	**Sequence change**	**Gene**	**Gene size**	**CDS position**	**Codon change**	**Amino acid change**	**Protein effect**
			bp			bp				
*S. macrospermum 302367*	2	Deletion	21	TCAAACAACCTTTTCGTAAAT	*rps18*	471	95	TTC, AAA, CAA, CCT, TTT, CGT, AAA, TCC → TCC	FKQPFRKS → S	Deletion
		Insertion	2	(T)3 → (T)5	*rpl22*	438	436	–	–	Frame shift
*S. macrospermum 326072*	2	Deletion	21	TCAAACAACCTTTTCGTAAAT	*rps18*	471	95	TTC, AAA CAA, CCT, TTT, CGT, AAA, TCC → TCC	FKQPFRKS → S	Deletion
		Insertion	2	(T)3 → (T)5	*rpl22*	438	436	–	–	Frame shift
*S. bicolor 314746*	1	Deletion	165	++	*rpoC2*	4398	2043	**	EDEYRTLEDEYETLEDEYGILEDEYRTLEKDSEEEYGSLENKYRTREGEGEYEILE → E	Deletion
*S. laxiflorum 326060*	1	Insertion	1	(T)10 → (T)11	*rps11*	432	431	–	–	Frame shift
*S. laxiflorum 326074*	1	Insertion	1	(T)10 → (T)11	*rps11*	432	431	–	–	Frame shift
*S. leiocladum 326061*	2	Insertion	21	TTTGTCTAATGATTAATTAAG	*matK*	1548	1540	–	–	Truncation
		Insertion	2	(T)10 → (T)11	*rps11*	432	431	–	–	Frame shift
*S. leiocladum 326062*	2	Insertion	21	TTTGTCTAATGATTAATTAAG	*matK*	1548	1540	–	–	Truncation
		Insertion	2	(T)10 → (T)11	*rps11*	432	431	–	–	Frame shift
*S. matarankense 326065*	3	Insertion	21	TTTGTCTAATGATTAATTAAG	*matK*	1548	1540	–	–	Truncation
		Insertion	5	TGGTT	*ycf2*	1987	1711	–	–	Frame shift
		Insertion	6	CACCAT	*ycf1*	813	712	→ CAC, CAT	→ HH	Insertion
*S. matarankense 326066*	3	Insertion	21	TTTGTCTAATGATTAATTAAG	*matK*	1548	1540	–	–	Truncation
		Insertion	5	TGGTT	*ycf2*	1987	1711	–	–	Frame shift
		Insertion	6	CACCAT	*ycf1*	813	712	→ CAC, CAT	→ HH	Insertion
*S. purpureosericeum 326068*	4	Insertion	21	TTTGTCTAATGATTAATTAAG	*matK*	1548	1540	–	–	Truncation
		Insertion	38	CACTGGGCGGAATGGGAAGACATAGTTAGTTATTCTTC	*rps3*	675	667	–	–	Truncation
		Insertion	5	TGGTT	*ycf2*	1987	1711	–	–	Frame shift
		Insertion	7	ATCCTAT	*ndhD*	1503	1500	–	–	Truncation
*S. purpureosericeum 326071*	4	Insertion	21	TTTGTCTAATGATTAATTAAG	*matK*	1548	1540	–	–	Truncation
		Insertion	38	CACTGGGCGGAATGGGAAGACATAGTTAGTTATTCTTC	*rps3*	675	667	–	–	Truncation
		Insertion	5	TGGTT	*ycf2*	1987	1711	–	–	Frame shift
		Insertion	7	ATCCTAT	*ndhD*	1503	1500	–	–	Truncation
*S. purpureosericeum 326075*	4	Insertion	21	TTTGTCTAATGATTAATTAAG	*matK*	1548	1540	–	–	Truncation
		Insertion	38	CACTGGGCGGAATGGGAAGACATAGTTAGTTATTCTTC	*rps3*	675	667	–	–	Truncation
		Insertion	5	TGGTT	*ycf2*	1987	1711	–	–	Frame shift
		Insertion	7	ATCCTAT	*ndhD*	1503	1500	–	–	Truncation
*S. brachypodum 302670*	4	Insertion	21	TTTGTCTAATGATTAATTAAG	*matK*	1548	1540	–	–	Truncation
		Insertion	2	(TT)5 → (TT)6	*rps11*	432	431	–	–	Frame shift
		Insertion	5	TGGTT	*ycf2*	1987	1711	–	–	Frame shift
		Insertion	6	CACCAT	*ycf1*	813	712	→ CAC, CAT	→ HH	Insertion
*S. brachypodum 326073*	4	Insertion	21	TTTGTCTAATGATTAATTAAG	*matK*	1548	1540	–	–	Truncation
		Insertion	4	(TT)5 → (TT)7	*rps11*	432	431	–	–	Frame shift
		Insertion	5	TGGTT	*ycf2*	1987	1711	–	–	Frame shift
		Insertion	6	CACCAT	*ycf1*	813	712	→ CAC, CAT	→ HH	Insertion

*Note*. Underlined text indicates the inserted or deleted sequence region.

++AGACGAATATAGGACTCTAGAGGACGAATATGAAACCCTAGAAGATGAGTATGGGATCCTAGAGGACGAATATAGGACTCTAGAGAAAGACTCAGAGGAAGAATATGGGAGCCTAGAGAACAAATATAGGACCCGAGAGGGAGAGGGCGAATATGAAATCCTAGA.

** GAA, GAC, GAA, TAT, AGG, ACT, CTA, GAG, GAC, GAA, TAT, GAA, ACC, CTA, GAA, GAT, GAG, TAT, GGG, ATC, CTA, GAG, GAC, GAA, TAT, AGG, ACT, CTA, GAG, AAA, GAC, TCA, GAG, GAA, GAA, TAT, GGG, AGC, CTA, GAG, AAC, AAA, TAT, AGG, ACC, CGA, GAG, GGA, GAG, GGC, GAA, TAT, GAA, ATC, CTA, GAG → GAG.

### 3.2 Chloroplast genome phylogeny

**FIGURE 4:**
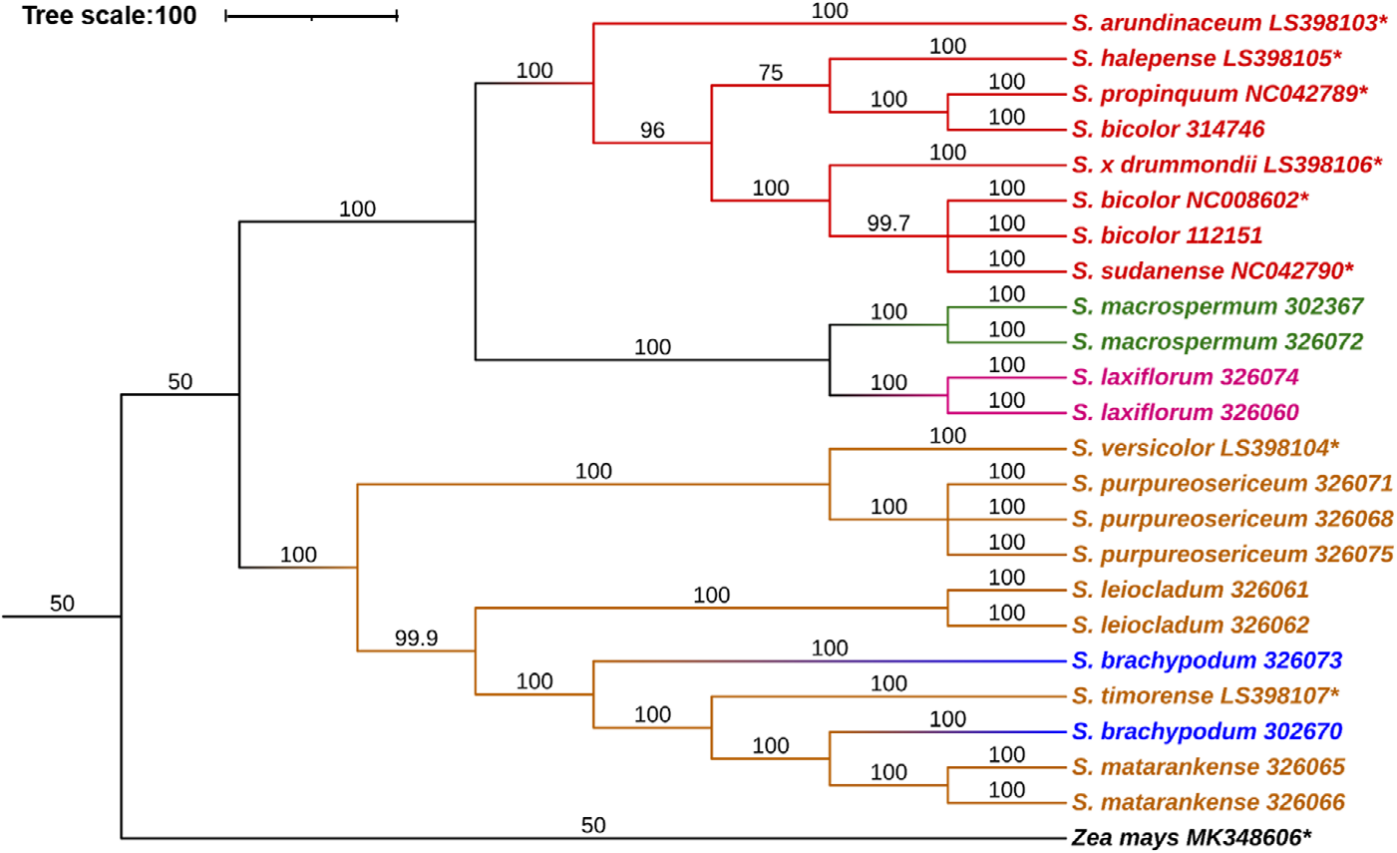
Chloroplast genome sequence‐based phylogeny of sorghum species. The phylogenetic tree of whole chloroplast genome sequences of 23 sorghum accessions with *Zea mays* L. as the outgroup. Tree was generated using maximum likelihood (ML) method in PAUP with 1,000 bootstrap replicates. Marked on each node is the ML bootstrap value (/100). Red: *Eusorghum*, Green: *Chaetosorghum*, Pink: *Heterosorghum*, Yellow: *Parasorghum*, Blue: *Stiposorghum*. *Sequences obtained from National Center for Biotechnology Information

The multiple chloroplast genome alignment of sorghum species together with the outgroup *Zea mays* was 142,647 bp in length with 96% identical sites. The optimal trees obtained by all three phylogenetic methods were topologically equivalent, and the final tree is presented in Figure 4. In the ML method, a strong bootstrap value (100%) was observed for the majority of nodes. The phylogenetic tree construction revealed two large distinct clades within these 23 sorghum accessions. Subgenera *Eusorghum*, *Chaetosorghum*, and *Heterosorghum* formed a single cluster, with *Stiposorghum* and *Parasorghum* positioned in a different cluster. All the *Eusorghum* species were resolved into one clade (*S. bicolor, S. propinquum, S. halepense, S. arundinaceum, S. sudanense*, and *S. *×* drummondii*). In this analysis, the *Eusorghum* clade becomes the sister group to the clades of *Heterosorghum* (*S. macrospermum 326072* and *302367*) and *Chaetosorghum* (*S. laxiflorum 326060* and *326074*). Within the *Eusorghum* clade, the accession *S. bicolor 314746* was resolved as a sister clade to *S. propinquum NC042789* rather than the other two *S. bicolor* accessions (*S. bicolor 112151* and *S. bicolor NC008602*). All the *Parasorghum* species (*S. leiocladum 326061* and *326062*; *S. matarankense 326065* and *326066*; *S. purpureosericeum 326068, 326071*, and *326075*; and *S. versicolor LS398104* and *S. timorense LS398107*) formed a single clade together with the *Stiposorghum* species *S. brachypodum 326073* and *302670*. However, the two accessions of *S. brachypodum 326073* and *302670* were not closely related to each other, being in two separate subclusters (Figure 4).

### 3.3 Nuclear genes phylogeny

**FIGURE 5:**
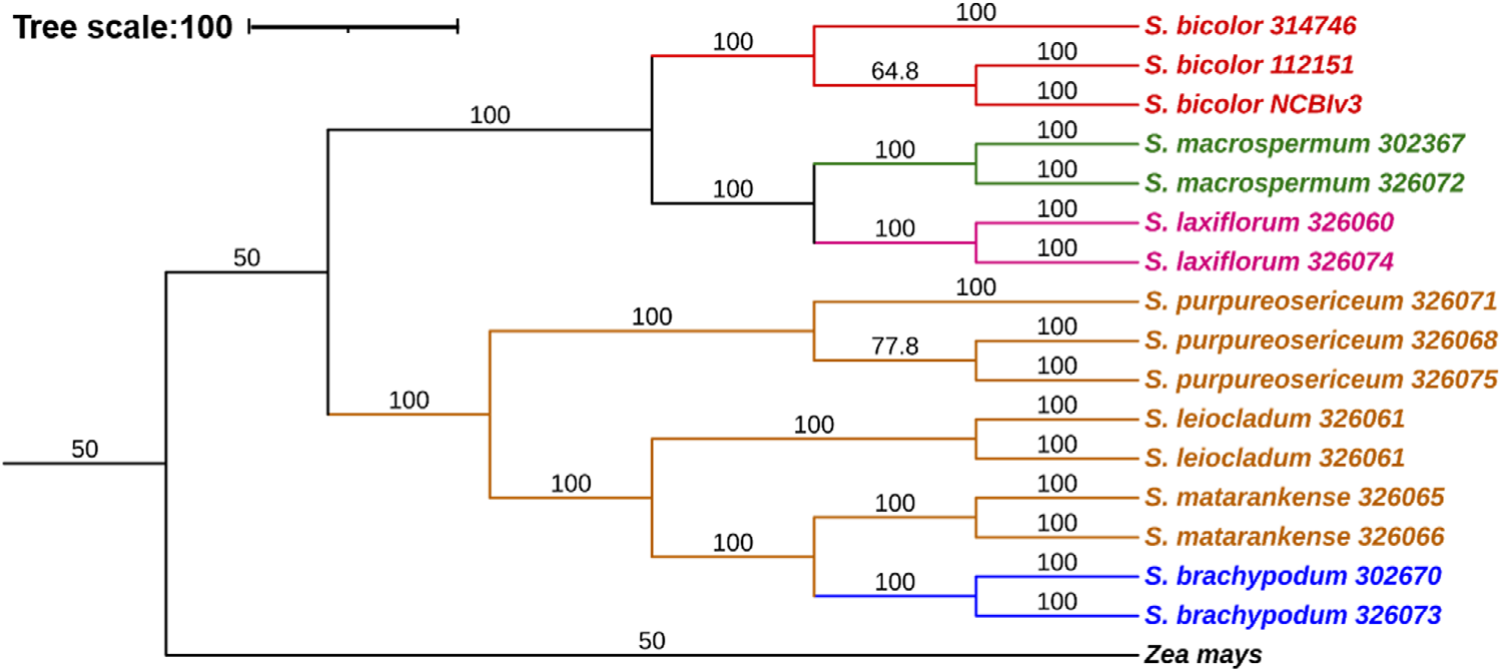
Selected nuclear genes‐based phylogeny of sorghum species. The phylogenetic tree of concatenated sequences of 99 single‐copy nuclear genes of 16 sorghum accessions, with *Zea mays* L. as the out group. Tree was generated using maximum likelihood (ML) method in PAUP with 1,000 bootstrap replicates. Marked on each node is the ML bootstrap value (/100). Red, *Eusorghum*; Green, *Chaetosorghum*; Pink, *Heterosorghum*; Yellow, *Parasorghum*; Blue, *Stiposorghum*

Generation of the nuclear genes phylogeny followed the same steps as used for the chloroplast phylogenetic analysis, with *Zea mays* as the outgroup. A total of 99 common single‐copy genes were selected based on the analysis of Li et al. (2017) and the multiple sequence alignment was 174,025 bp in length with 76.7% identical sites. The optimal trees obtained by all three phylogenetic methods were topologically equivalent and the final tree is presented in Figure 5. In the ML method, a strong bootstrap value of 100% was observed for the majority of the nodes. According to the phylogenetic tree generated in this analysis, two distinct clusters were identified among these 16 sorghum accessions, which mimics the chloroplast genome phylogeny. Subgenera *Eusorghum*, *Chaetosorghum*, and *Heterosorghum* localized in a separate cluster and *Stiposorghum* and *Parasorghum* in a separate cluster. All the *Eusorghum* species formed a single clade, which is a sister clade to the clades of *Chaetosorghum* and *Heterosorghum*. All the *Parasorghum* species were in one clade together with the *Stiposorghum* species *S. brachypodum 326073* and *302670*. However, unlike in the chloroplast phylogeny, the two *S. brachypodum* species were clustered together in a separate subclade.

## 4 DISCUSSION

Phylogenetic analysis based on organelle genomes play a vital role in determining relationships among closely related species (Carbonell‐Caballero et al., 2015; Dong et al., 2017). At the species level, it can be challenging to resolve phylogenetic relationships based on a small number of loci (Hilu & Alice, 2001; Majure et al., 2012). Studies on phylogenetic relationships, especially among the angiosperms, have been improved with the use of complete chloroplast genome sequencing (Bausher et al., 2006; Cai et al., 2006; Chang & Lin, 2006).

With the recent advances of next generation sequencing technology, more efficient and cost‐effective methods are available. In this study, next generation sequencing was used to sequence and assemble the complete chloroplast genomes of wild relatives of the important crop *S. bicolor*. The accuracy of the assembled chloroplast genomes is strongly influenced by the choice of assembly pipeline (Sims et al., 2014). Here we used two assembly approaches, reference‐guided mapping assembly and de novo assembly, followed by comparison of the assemblies to identify and resolve discrepancies for improved accuracy of the final assembled chloroplast genome sequence. The typical structure of the chloroplast, with the same gene content and gene order, was observed for all the species.

Different methods are available to annotate the chloroplast genome such as GeSeq (Tillich et al., 2017), Dogma (Wyman et al., 2004), PLANN (Plastome Annotator) (Huang & Cronk, 2015), CGAP (Chloroplast Genome Analysis Platform) (Cheng et al., 2013), CpGAVAS (Chloroplast Genome Annotation, Visualization, Analysis, and GenBank Submission) (Liu et al., 2012). However, many of these tools are considered outdated due to drawbacks such as low accuracy. The efficiency of GeSeq compared with Dogma has been assessed in the study by Guyeux et al. (2019), indicating that GeSeq is the best available tool for chloroplast genome annotation. In the present study, GeSeq software was used to annotate the chloroplast genomes, allowing the annotation of more genes compared with the previous study of Song et al. (2019) (77 protein‐coding genes, 29 tRNA genes, and 4 rRNA genes).

The mutations, which are clustered as hotspots, have created highly variable regions in the chloroplast genomes which are very informative in phylogenetic analysis (Dong et al., 2012). In the current study, three main sequence variant hotspots were identified (*rpoC2, matK*, and *ndhF*). Studies have identified the *rpoC2* gene as a highly variable region, and this has been used successfully in phylogenetic studies in the family Poaceae (Cummings et al., 1994; Barker et al., 1999 ; Duvall et al., 2001; Moon et al., 2016). Higher frequencies of SNPs and Indels have also been identified in *matK* and *ndhF* in citrus (*Citrus* spp.) and were shown to be useful for phylogenetic analysis (Carbonell‐Caballero et al., 2015). In addition, the *rps15‐ndhF* region has been successfully used in phylogenetic studies in grasses (Zhang et al., 2016). The study of Song et al. (2019) identified mutational hotspot regions, including *rps15*‐*ndhF* as well as *rps16‐trnQ*, *trnG‐trnM*, and *rbcL‐psaI*, in chloroplast genomes of sorghum by sliding window analysis. The differences in identification of regions of high variation within the sorghum chloroplast genome may be because in the current study we have analyzed the sequence from a greater number of species using more accurate sequencing technology and improved annotation. It has also been suggested that the regions of high variation in *matK* and *ndhF* are due to environmental pressures and may be linked to the adaptation of plants to hot and dry conditions (Daniell et al., 2016).

Indels in coding sequences were identified in genes *rpl22*, *rps11*, and *ycf2*, which caused frameshifts in the resulting protein. The accuracy of these variants was checked by examining the mapping files of these final sequences. Either there were no conflicts or if there were conflicts, the majority call for the base in the reads was used in the consensus sequences (Supplemental Table S5). Indels, not representing multiples of three bases, lead to a frameshift causing a change in amino acid sequence from the point of the variant insertion (Table 4). Proteins with these variants might have lost their function in the chloroplast. Some studies have reported that copies of some of these above genes have been translocated to other genomes within the cell. For instance, the gene *rpl*22, encodes the chloroplast ribosomal protein, and a functional copy of this gene has been reported to be located, not in the chloroplast, but in the nucleus in the *Pisum sativum* L. (Gantt et al., 1991), and putative losses of this gene has been reported in Fagaceae and Passifloraceae plants (Jansen et al., 2011). Horizontal gene transfer events have been reported for the gene *rps11* where mitochondrial genomes have acquired a copy of this gene in the families Betulaceae, Caprifoliaceae, and *Sanguinaria* (Richardson & Palmer, 2007). The gene *ycf2* is considered as a pseudogene, and loss of the *ycf2* gene in the plastid genome has been reported in the Poaceae family concomitant with the absence of a copy in the nucleus (Wicke et al., 2011; Downie & Palmer, 1992). In addition, truncation of the sequences of the three genes *matK*, *rps3*, and *ndhD* has been observed in *S. leiocladum*, *S. matarankense*, *S. purpureosericeum*, and *S. brachypodum*. Studies by Xu et al. (2015) and Wicke et al. (2011) suggested that the genes *matK*, *ndhD*, *rpl22*, *rps11*, and *ycf2* were lost from chloroplast genomes of land plants in the process of evolution. Future studies are required to determine whether these proteins are still functional or not in *Sorghum*.

In the phylogenetic analysis, a similar tree topology was observed for both the chloroplast and nuclear gene phylogeny, with only minor differences in the subgenus *Stiposorghum*. Interestingly, in the chloroplast phylogeny, the two accessions of *S. brachypodum 326073* and *302670* did not form a monophyletic group but were instead paraphyletic with two species *S. timorense* and *S. matarankense*. These two *S. brachypodum* accessions have originated from two different wild populations. *S. brachypodum 326073* is a single seed descendent line derived from *302481* and collected from a natural wild population west of Jabiru, Kakadu National Park, NT, Australia (geocoordinates: 12.7145 "S, 132.4167 "E). *S. brachypodum 302670* is a single plant grown from a natural population collected from a population near the Oenpelli Road northeast of Jabiru, Kakadu NP (geocoordinates: 12.5667 ″S, 132.88 ″E). However, these two accessions showed similar nuclear genes confirming their identity as a single species. The polyphyletic origins of their chloroplast genomes indicate there has been some degree of gene flow between the species. Chloroplast transfer or capture is common in closely related plant species and represents a clear example of reticulate evolution. Whole chloroplast genome sequencing has revealed this process in many plant groups (Guyeux et al., 2019; Healey et al., 2018; Moner et al., 2020). This analysis shows that *Stiposorghum* and *Parasorghum* are not monophyletic groups.

A further example of discordant chloroplast phylogeny was found within the *Eusorghum* clade. The accession *S. bicolor 314746* was a sister group to *S. propinquum NC042789* rather than to the other two *S. bicolor* accessions, *S. bicolor 112151* and *S. bicolor NC008602*. The close relationship between *S. propinquum* and *S. bicolor* could be be a result of *S. propinquum* being a perennial rhizomatous form of *S. bicolor* (Chittenden et al., 1994; Sun et al., 1994). Furthermore, both the reference *S. bicolor NC008602* and *S. bicolor 112151* are different accessions from different seed sources of the same cultivar Tx623 B (maintainer) whereas *S. bicolor 314746* is from the cultivar Tx623 A (male sterile) (Table 1), which are breeding lines of a single variety of Tx623. Recent studies revealed variations within chloroplast genome sequences (Moner et al., 2018, 2020) that could be associated with multiple domestication events in rice (*Oryza sativa* L.). Although the nuclear phylogeny places the *S. bicolor* accessions in one clade, this does not exclude the possibility that more than one chloroplast type from wild progenitors has been captured by domesticated sorghum as has been found in rice (Moner et al., 2020). Multiple domestications or chloroplast capture by reticulate evolution (Jackson et al., 1999) may be involved. Future studies targeting more species within the groups *Eusorghum* and *Stiposorghum* are required to resolve the poorly defined phylogeny within these subgenera. In both phylogenetic trees, two major clades were observed suggesting that the genus *Sorghum* can be reclassified into two major subgenera including *Eusorghum*, *Chaetosorghum*, and *Heterosorghum* in one group and *Parasorghum* and *Stiposorghum* in the other group. The classification into two clades confirms the findings of Dillon et al. (2007a).

Despite some contradicting data on sorghum classification, distinct trends have emerged and are being substantiated (Sun et al., 1994; Dillon et al., 2001; Dillon et al., 2004; Dillon et al., 2007a; Liu et al., 2014). Dillon et al. (2007a), for example, determined all the *Eusorghum* species to be in the *Eusorghum* clade with strong bootstrap values. This was based on combined sequencing data of *Adh1*, *ITS1*, and *ndhF* from all species within the genus (including *S. exstans, S. intrans, S. angustum, S. interjectum, S. ecarinatum, S. stipoideum, S. amplum, S. bulbosum, S. plumosum, S. grande*, and *S. nitidum*). The study of Liu et al. (2014) revealed the same trend in phylogenetic tree constructions. The nuclear genes *Pepc4* and *GBSSI*, as well as *ndhA*, *rpl32*‐*trnL*, and *rps16* introns, in chloroplast genes resulted in three clusters, including *Eusorghum* lineage, *Chaetosorghum* and *Heterosorghum* lineage, and *Parasorghum* and *Stiposorghum* lineage. Studies by Dillon et al. (2007a) also supported an earlier proposal to combine the two monotypic subgenera: *Chaetosorghum* and *Heterosorghum* into one group (Spangler, 2003) within the group *Eusorghum*. These two species have some similarities in morphology, such as minute obtuse callus (Lazarides et al., 1991), and are also cytologically very similar to each other but distinct from other Australian sorghum species (Dillon et al., 2007a; Lazarides et al., 1991; Price et al., 2005).

Spangler (2003) combined *Stiposorghum* and *Parasorghum* into a new genus, *Sarga*, based on morphological and molecular data. Our chloroplast and nuclear sequence‐based phylogeny analyses place the subgenera *Stiposorghum* and *Parasorghum* in one well‐supported clade with very strong bootstrap values consistent with the phylogeny presented by Dillon et al. (2007a). A study by Lazarides et al. (1991) differentiated *Parasorghum* from *Stiposorghum* using the structural features of the callus and articulation joint of the sessile spikelet. This approach was questioned in the later study of Spangler (2003). The species in the clade *Stiposorghum* were not fully resolved by Dillon et al. (2007a). By contrast, we were able to fully resolve all species in both these two subgenera, again well supported with strong bootstrap values. Moreover, in the chloroplast phylogeny, the two species *S. purpureosericeum* and *S. versicolor*, which are of African origin, were determined to be closely related to each other supporting the findings of Dillon et al. (2007a). However, a comprehensive analysis including all the species in the genus is required to fully support our results.

Compared with other major cereal crops, the knowledge of plant growth and developmental diversity in the wild relatives of sorghum is scarce (Cowan et al., 2021; Dillon et al., 2007b; Kuhlman et al., 2010; Lazarides et al., 1991). Of special interest is the need to understand possible retained beneficial traits in the wild sorghum relatives offering improved resilience to environmental stresses (Cowan et al., 2020; Myrans et al., 2021). Drought stress is a key factor limiting crop yield at the global scale and its effect on yield loss is expected to be aggravated by climate change. In a recent study (Cowan et al., 2020), the growth, morphological, physiological, and biochemical characteristics of seven wild *Sorghum* species belonging to the subgenera *Chaetosorghum*, *Parasorghum*, and *Stiposorghum* were compared with that of *S. bicolor*. All seven wild sorghum species showed a significantly reduced level of the toxic cyanogenic glucoside dhurrin in the leaves (Gleadow & Møller, 2014; Pičmanová et al., 2015). Two close relatives of domesticated sorghum showed distinct traits. *Sorghum brachypodum* was the least affected by drought stress and its content of toxic dhurrin was low (1 μg g^−1^ tissue) and not induced by drought stress. In the elite cultivar *S. bicolor*, the corresponding dhurrin levels were 500‐ and 1,000‐fold higher (Cowan et al., 2020). *Sorghum macrospermum* produced greater biomass compared with *S. brachypodum* but was more affected by drought. Given that that these two species are in the same clade as *S. bicolor*, and therefore closely related phylogenetically, there may be an advantage in breeding efforts to transfer the trait of acyanogenic leaves to elite *S. bicolor* cultivars. The phylogenetic analyses provided may guide the selection of other wild sorghum relatives with interesting growth features to be introduced into elite sorghum cultivars for the future.

## 5 CONCLUSION

Complete chloroplast genome and concatenated nuclear gene analyses were carried out using a total of 15 accessions representing five subgenera of the genus *Sorghum*. The phylogenetic analyses based on complete chloroplast and nuclear gene sequences resulted in highly similar phylogenetic trees. The genus *Sorghum* can be divided into two main groups, including *Eusorghum*, *Chaetosorghum*, and *Heterosorghum* in one group and *Parasorghum* and *Stiposorghum* in the other group. However, a complete and comprehensive study including all the species in the genus is required to accurately define the relationships within the genus *Sorghum*.

## AUTHOR CONTRIBUTIONS

**Robert J. Henry**: Conceptualization, Methodology, Supervision, Project administration, Funding acquisition, Resources and Writing ‐ Review & Editing. **Roslyn Gleadow**: Conceptualization, Supervision, Funding acquisition, Writing ‐ Review & Editing. **Birger Lindberg Møller**: Conceptualization, Funding acquisition, Review & Editing. **Agnelo Furtado**: Methodology, Formal analysis, Supervision, Data Curation, Writing ‐ Review & Editing. **Sally L. Norton**: Methodology, Resources and Writing ‐ Review & Editing. **Cecilia Blomstedt**: Methodology, Data collection, Resources and Writing ‐ Review & Editing. **Galaihalage K. S. Ananda**: Formal analysis, Data Curation, Investigation, Writing ‐ Original Draft.

## CONFLICT OF INTEREST

The authors declare no conflict of interest.

## Supporting information


**Supplemental Table S1**: Details of the illumine sequencing data of the sorghum samples.
**Supplemental Table S2**: Details of the 99 single copy genes used in this study (Li et al., 2017) for the nuclear gene phylogenetic tree construction.
**Supplemental Table S3**: Parameters of the substitution model selected from the jModelTest2 for chloroplast phylogeny.
**Supplemental Table S4**: Parameters of the substitution model selected from the jModelTest2 for nuclear phylogeny.
**Supplemental Table S5**: Details of the Indels inside CDS causing frameshifts and truncations of the proteins are given.

## Data Availability

All data and materials used and described in this study are made available for noncommercial research purposes. The data that support the findings of this study are openly available in Sequence Read Archive (SRA) under the BioProject number PRJNA692754.
