# Supplementary material for: Phylogenetic relationships in the Sorghum genus based on sequencing of the chloroplast and nuclear genes
Source: Plant Genome. 2021 Jul 29;14(3):e20123. doi: 10.1002/tpg2.20123 (PMC12807248; doi:10.1002/tpg2.20123)
Supplement: Supplementary file 1 [file TPG2-14-e20123-s001.pdf]

Phylogenetic relationships in the Sorghum genus based on sequencing of the chloroplast and nuclear genes

Galaihalage K. S. Ananda[1], Sally L. Norton[2], Cecilia Blomstedt[3], Agnelo Furtado[1], Birger Lindberg Møller[4], Roslyn Gleadow[1,3] and Robert J. Henry[1*]

[1] Queensland Alliance for Agriculture and Food Innovation, The University of Queensland, St Lucia, QLD, Australia,

[2] Australian Grains Genebank, Agriculture Victoria, Horsham, Vic, Australia,

[3] School of Biological Sciences, Monash University, Clayton, Vic, Australia,

[4] Plant Biochemistry Laboratory, Department of Plant and Environmental Sciences, University of Copenhagen, Copenhagen, Denmark

Correspondence:

Robert J. Henry, Queensland Alliance for Agriculture and Food Innovation, The University of Queensland, St Lucia, QLD, Australia

robert.henry@uq.edu.au

Number of pages: 21

Number of tables: 05

Supplemental Table S1: Details of the illumine sequencing data of the sorghum samples.

DNA-Seq file ID	Genotype	Coverage	Total number of raw reads	Total number of bases	Total number of 0.01 reads	% Loss 0.01 reads	Total number of 0.01 bases	% Loss 0.01 bases
SorgMacr326072	*S. macrospermum*	20X	195,842,812	29,285,527,035	195,840,759	0.001	26,724,242,067	8.75
SorgMac302367	*S. macrospermum*	36X	284,740,322	42,709,187,715	276,109,890	3.000	40,020,617,954	6.30
SorgBic314746	*S. bicolor*	17X	92,218,048	13,817,750,058	89,650,414	3.000	13,038,912,045	5.64
SorgBic112151	*S. bicolor*	12X	68,805,710	10,323,586,958	66,638,305	3.000	9,706,710,308	5.98
SorgLax326060	*S. laxiflorum*	30X	224,988,548	33,702,293,833	217,147,944	3.000	31,559,914,062	6.36
SorgLax326074	*S. laxiflorum*	18X	164,082,972	24,776,528,772	157,677,240	3.903	23,122,097,964	6.67
SorgLeio326061	*S. leiocladum*	20X	430,767,338	64,455,151,632	430,766,441	0.000	57,335,492,671	11.05
SorgLei326062	*S. leiocladum*	32X	392,874,086	58,879,335,621	380,773,774	3.000	55,478,641,600	5.78
SorgMata326065	*S. matarankense*	20X	188,321,560	28,172,194,786	188,320,327	0.001	25,540,575,330	9.34
SorgMat326066	*S. matarankense*	24X	301,612,284	45,237,951,580	290,225,262	4.000	42,006,884,224	7.14
SorgPurp326068	*S. purpureosericeum*	20X	388,072,484	57,847,395,472	388,069,263	0.001	52,891,826,749	8.57
SorgPur326071	*S. purpureosericeum*	24X	441,210,716	66,138,955,235	427,721,502	3.000	62,222,909,126	5.92
SorgPur326075	*S. purpureosericeum*	25X	537,816,756	80,612,846,813	516,800,277	4.000	74,895,565,130	7.09
SorgBra326073	*S. brachypodum*	16X	214,759,700	32,178,794,016	204,068,980	5.000	29,319,866,897	8.88
SorgBra302670	*S. brachypodum*	36X	311,748,290	46,740,864,578	301,192,220	3.000	43,792,111,919	6.31

Supplemental Table S2: Details of the 99 single copy genes used in this study (Li et al., 2017) for the nuclear gene phylogenetic tree construction.

	Protein name	*Sorghum bicolor*		*Zea mays*	
		**Gene symbol**	**blast description (Gene)**	**Gene symbol**	**blast description (Gene)**
01	4-alpha-glucanotransferase DPE2	LOC8072271	4-alpha-glucanotransferase DPE2	LOC100194185	uncharacterized LOC100194185
02	50S ribosome binding GTPase	LOC8085029	obg-like ATPase 1	LOC100283841	GTP-binding protein PTD004
03	A49-like RNA polymerase I associated factor	LOC8067921	uncharacterized LOC8067921	LOC100284469	uncharacterized LOC100284469
04	Aconitase (PF00330)	LOC8085012	putative aconitate hydratase	LOC103647708	putative aconitate hydratase
05	Acyl transferase domain	LOC8081118	malonyl CoA-acyl carrier protein transacylase	LOC100283964	uncharacterized LOC100283964
06	Acyl-CoA dehydrogenase, C-terminal domain	LOC8071258	isovaleryl-CoA dehydrogenase	LOC100382372	uncharacterized LOC100382372
07	ALAD (PF00490) Porphobilinogen synthase	LOC110431267	Delta-aminolevulinic acid dehydratase	LOC100284052	Delta-aminolevulinic acid dehydratase
08	Aldoketo reductase family	LOC8063259	L-galactose dehydrogenase	LOC732776	L-galactose dehydrogenase (GalDH) mRNA
09	Alphabeta hydrolase family	LOC110430194	2-hydroxy-6-oxononadienedioate/2-hydroxy-6-oxononatrienedioate hydrolase	LOC100285951	uncharacterized LOC100285951
10	Amino acid permease	LOC8073963	cationic amino acid transporter 9	LOC100285703	yfnA
11	Aminomethyltransferase folate-binding domain	LOC8066805	aminomethyltransferase, mitochondrial	LOC100279332	uncharacterzed LOC100279332
12	Aminopeptidase I zinc metalloprotease (M18)	LOC8074128	probable aspartyl aminopeptidase	LOC100304311	Zn-dependent exopeptidase superfamily protein
13	ArgJ family	LOC8081475	arginine biosynthesis bifunctional protein ArgJ	LOC100383335	Arginine biosynthesis bifunctional protein ArgJ
14	Armadillobeta-catenin-like repeat	LOC8059116	importin subunit *alpha 2*	LOC100281383	importin alpha-2 subunit
15	ATP synthase delta (OSCP) subunit	LOC8071801	ATP synthase subunit O	LOC100282837	ATP synthase subunit O
16	Brix domain	LOC8080081	ribosome production factor 2 homolog	LOC100282857	clone 243603 brix domain-containing protein 1 mRNA
17	Cactus-binding C-terminus of cactin protein	LOC8061158	Cactin	LOC100382147	Cactin

18	Carbon-nitrogen hydrolase	LOC8059630	N-carbamoylputrescine amidase	LOC100282130	clone 287777 hydrolase, carbon-nitrogen family mRNA
19	CLASP N terminal	LOC8061883	CLIP-associated protein	LOC103646242	CLIP-associated protein
20	CobWHypBUreG, nucleotide-binding domain	LOC8074601	urease accessory protein G	LOC100217046	clone 1557664 urease accessory protein ureG mRNA
21	Cohesin loading factor	LOC8069436	uncharacterized LOC8069436	LOC100192084	uncharacterized LOC100192084
22	Copper amine oxidase, enzyme domain	LOC8073581	copper methylamine oxidase	LOC100383438	uncharacterized LOC100383438
23	CreatinaseProlidase N-terminal domain	LOC8063695	probable Xaa-Pro aminopeptidase P	LOC100381889	uncharacterized LOC100381889
24	Cullin family	LOC8084141	cullin-4	LOC103625822	cullin-4
25	Cyclophilin type peptidyl-prolyl cis-trans isomeraseCLD	LOC8074358	peptidyl-prolyl cis-trans isomerase CYP23	LOC100193083	Peptidyl-prolyl cis-trans isomerase CYP23
26	Cytochrome b5-like HemeSteroid binding domain	LOC110433021	uncharacterized LOC110433021	LOC100272407	uncharacterized LOC100272407
27	DDRGK domain	LOC8081581	DDRGK domain-containing protein 1	LOC100272547	uncharacterized LOC100272547
28	Dienelactone hydrolase family	LOC8074965	protein usf	LOC100282241	Alpha/beta-Hydrolases superfamily protein
29	Dihydroorotate dehydrogenase	LOC8068612	dihydropyrimidine dehydrogenase (NADP (+))	LOC100274265	uncharacterized LOC100274265
30	Divergent CRALTRIO domain	LOC8081330	protein GDAP2 homolog	LOC100282379	uncharacterized LOC100282379
31	DnaJ domain	LOC8054304	J domain-containing protein spf31	LOC100282966	Chaperone DnaJ-domain superfamily protein
32	Dual specificity phosphatase, catalytic domain	LOC8066946	phosphoglucan phosphatase LSF2	LOC732726	clone 399764 dual-specificity protein-like phosphatase 3 mRNA
33	Dynamin family (2)	LOC8082754	dynamin-like protein ARC5	LOC100383294	uncharacterized LOC100383294
34	Dynamin family	LOC8068607	dynamin-2A	LOC100286124	dynamin-2A
35	Elongation factor Tu GTP binding domain	LOC8061800	translation initiation factor IF-2	LOC103630286	PREDICTED: translation initiation factor IF-2
36	ER membrane protein complex subunit 1, C-terminal	LOC8058276	ER membrane protein complex subunit 1	LOC103635715	ER membrane protein complex subunit 1-like
37	Eukaryotic protein of unknown function (DUF866)	LOC8078606	UPF0587 protein F46B6.12	LOC100276801	uncharacterized LOC100276801
38	FAD binding domain	LOC110435654	L-galactono-1,4-lactone dehydrogenase 2	LOC100381436	uncharacterized LOC100381436

39	GcpE protein	LOC8072432	4-hydroxy-3-methylbut-2-en-1-yl diphosphate synthase (ferredoxin)	LOC542690	full-length cDNA clone ZM_BFb0029N16 mRNA
40	Glucose-6-phosphate isomerase	LOC8083248	glucose-6-phosphate isomerase 1	LOC100281592	glucose-6-phosphate isomerase
41	Glycosyl hydrolases family 31	LOC8056883	probable glucan 1,3-alpha-glucosidase	LOC100383593	putative glucan 13-alpha-glucosidase
42	GlyoxalaseBleomycin resistance proteinDioxygenase superfamily	LOC8071534	uncharacterized protein YwkD	LOC100283949	uncharacterized LOC100283949
43	GMP synthase C terminal domain	LOC8076514	GMP synthase [glutamine-hydrolyzing]	LOC100383569	uncharacterized LOC100383569
44	haloacid dehalogenase-like hydrolase	LOC8062162	uncharacterized LOC8062162	LOC100280165	uncharacterized LOC100280165
44	HCNGP-like protein	LOC8079227	serine/arginine repetitive matrix protein 1	LOC103636629	Putative HCNGP-related transcriptional regulator family protein
46	Histidinol dehydrogenase	LOC110431479	histidinol dehydrogenase	LOC100284964	uncharacterized protein LOC100284964
47	Hydroxyacylglutathione hydrolase C-terminus	LOC8083670	hydroxyacylglutathione hydrolase	LOC100193826	uncharacterized LOC100193826
48	Inositol monophosphatase family	LOC8085818	bifunctional phosphatase IMPL2	LOC100280816	inositol-1-monophosphatase
49	Integral membrane protein DUF92	LOC8062116	protein VTE6	LOC100283913	putative conserved membrane protein
50	Lon protease family	LOC8077511	lon protease homolog	LOC732810	LON2 protein
51	Memo-like protein	LOC8062333	protein MEMO1	LOC100273137	uncharacterized LOC100273137
52	Methylenetetrahydrofolate reductase	LOC8057390	methylenetetrahydrofolate reductase 1	LOC541794	methylenetetrahydrofolate reductase mRNA
53	Methyltransferase domain	LOC8073167	probable tocopherol O-methyltransferase	LOC732837	clone 245696 tocopherol O-methyltransferase mRNA
54	MINDY deubiquitinase	LOC8064982	ubiquitin carboxyl-terminal hydrolase MINDY-1	LOC103629574	ubiquitin carboxyl-terminal hydrolase MINDY-1
55	Molybdopterin oxidoreductase	LOC8063381	NADH dehydrogenase [ubiquinone] iron-sulfur protein 1	LOC100191453	NADH-ubiquinone oxidoreductase subunit
56	Nitrite and sulphite reductase 4Fe-4S domain	LOC8080077	sulfite reductase [ferredoxin]	LOC542221	sulfite reductase1
57	NLI interacting factor-like phosphatase	LOC8063118	ubiquitin-like domain-containing CTD phosphatase	LOC100282666	uncharacterized LOC100282666

58	NmrA-like family	LOC8055309	NADH dehydrogenase [ubiquinone] 1 alpha subcomplex subunit 9	LOC100282690	uncharacterized LOC100282690
59	N-terminal domain of lipoyl synthase of Radical_SAM family	LOC8057459	lipoyl synthase	LOC100279807	Lipoyl synthase
60	Paf1	LOC8069621	protein PAF1 homolog	LOC100279273	uncharacterized LOC100279273
61	PAP2 superfamily C-terminal	LOC8057075	uncharacterized LOC8057075	LOC100277618	uncharacterized LOC100277618
62	Peroxidase	LOC8058035	probable L-ascorbate peroxidase 4	LOC100193965	clone 688596 APx4 - Peroxisomal Ascorbate Peroxidase
63	Plant specific mitochondrial import receptor subunit TOM20	LOC8065688	protein HLB1	LOC100274434	uncharacterized LOC100274434
64	PROCN (NUC071) domain	LOC8070895	pre-mRNA-processing-splicing factor 8A	LOC103638016	pre-mRNA-processing-splicing factor 8A
65	Prolyl oligopeptidase, N-terminal beta-propeller domain	LOC8069244	protease 2	LOC100281516	protease 2
66	Protein prenyltransferase alpha subunit repeat	LOC8054810	protein farnesyltransferase/geranylgeranyltransferase type-1 subunit alpha	LOC100273635	protein farnesyltransferase/geranylgeranyltransferase type I alphasubunit
67	Putative tRNA binding domain	LOC8071735	probable methionine--tRNA ligase	LOC103654058	probable methionine--tRNA ligase
68	Pyruvate phosphate dikinase, AMPATP-binding domain	LOC8085351	alpha-glucan water dikinase	LOC100381411	Alpha-glucan water dikinase 1
69	R3H-associated N-terminal domain	LOC8085655	uncharacterized LOC8085655	LOC100276056	uncharacterized LOC100276056
70	Redoxin	LOC8074509	peroxiredoxin-2F	LOC100273860	peroxiredoxin-5
71	Respiratory-chain NADH dehydrogenase 51 Kd subunit	LOC110432457	NADH dehydrogenase [ubiquinone] flavoprotein 1	LOC100282384	NADH-ubiquinone oxidoreductase 51 kDa subunit
72	Ribophorin I	LOC8061440	dolichyl-diphosphooligosaccharide--protein glycosyltransferase subunit 1A	LOC100285254	dolichyl-diphosphooligosaccharide--protein glycosyltransferase 67 kDa subunit
73	Ribosomal protein L13	LOC8072074	50S ribosomal protein L13	LOC100192010	50S ribosomal protein L13
74	RNA recognition motif. (a.k.a. RRM, RBD, or RNP domain)	LOC8069812	SUPPRESSOR OF ABI3-5	LOC103636240	SUPPRESSOR OF ABI3-5
75	Rubrerythrin	LOC8074544	magnesium-protoporphyrin IX monomethyl ester [oxidative] cyclase	LOC103634652	magnesium-protoporphyrin IX monomethyl ester [oxidative] cyclase,

76	Scaffold protein NfuNifU N terminal	LOC8069538	nifU-like protein 4	LOC100192708	uncharacterized LOC100192708
77	Sec1 family	LOC8066458	SEC1 family transport protein SLY1	LOC100280268	SEC1-family transport protein SLY1
78	Sec23Sec24 trunk domian	LOC8080287	protein transport protein Sec24-like At3g07100	LOC100194158	uncharacterized LOC100194158
79	SHNi-TPR	LOC8059878	protein HGV2	LOC101027138	TPR domain containing protein
80	snoRNA binding domain, fibrillarin	LOC8055974	U4/U6 small nuclear ribonucleoprotein Prp31 homolog	LOC100194058	pre-mRNA processing ribonucleoprotein binding region-containing protein
81	Sodium Bile acid symporter family	LOC8054950	probable sodium/metabolite cotransporter BASS2	LOC100303815	bile acid sodium symporter
82	Staphylococcal nuclease homologue	LOC8056813	ribonuclease TUDOR 1	LOC103627215	ribonuclease TUDOR 1
83	SYF2 splicing factor	LOC8065609	pre-mRNA-splicing factor syf2	LOC100272811	uncharacterized LOC100272811
84	TATA-binding protein interacting (TIP20)	LOC8074696	cullin-associated NEDD8-dissociated protein 1	LOC100383873	uncharacterized LOC100383873
85	TFIIS helical bundle-like domain	LOC8060802	protein IWS1 homolog 1	LOC100282789	IWS1 C-terminus family protein
86	Thiamine pyrophosphate enzyme, N-terminal TPP binding domain	LOC8056536	2-hydroxyacyl-CoA lyase	LOC100191888	2-hydroxyphytanoyl-CoA lyase
87	Thioredoxin-like [2Fe-2S] ferredoxin	LOC110430556	NADH dehydrogenase [ubiquinone] flavoprotein 2	LOC100272681	NADH dehydrogenase [ubiquinone] flavoprotein 2
88	Transcription factor TFIIB repeat	LOC8054252	plant-specific TFIIB-related protein 1	LOC542022	partial mRNA for TFIIB-related protein
89	tRNA N6-adenosine threonylcarbamoyltransferase	LOC8065902	probable tRNA N6-adenosine threonylcarbamoyl transferase	LOC100273984	O-sialoglycoprotein endopeptidase
90	tRNA synthetase class II core domain (G, H, P, S and T)	LOC8058371	threonine--tRNA ligase	LOC103626016	threonine--tRNA ligase,
91	tRNA synthetases class I (E and Q), catalytic domain (2)	LOC8078747	glutamine--tRNA ligase	LOC100285944	glutaminyl-tRNA synthetase
92	tRNA synthetases class I (E and Q), catalytic domain	LOC8065086	110 kDa U5 small nuclear ribonucleoprotein component CLO	LOC103638709	116 kDa U5 small nuclear ribonucleoprotein component-like
93	tRNA synthetases class I (I, L, M and V)	LOC8084035	leucine--tRNA ligase	LOC100282419	uncharacterized LOC100282419
94	tRNA synthetases class I (W and Y)	LOC8066308	tryptophan--tRNA ligase	LOC103645798	leucine--tRNA ligase

95	Ubiquitin carboxyl-terminal hydrolase, family 1	LOC8057798	ubiquitin carboxyl-terminal hydrolase 2	LOC100382000	uncharacterized LOC100382000
96	Ubiquitin carboxyl-terminal hydrolase	LOC8075479	ubiquitin carboxyl-terminal hydrolase 6	LOC100282385	ubiquitin carboxyl-terminal hydrolase 6
97	Universal stress protein family	LOC8054981	uncharacterized LOC8054981	LOC100193714	universal stress family protein
98	WLM domain	LOC8067004	uncharacterized LOC8067004	LOC100275609	uncharacterized LOC100275609
99	Zinc-finger of C2H2 type	LOC8054294	splicing factor 3A subunit 2	LOC100272589	Splicing factor 3A subunit 2

Supplemental Table S3: Parameters of the substitution model selected from the jModelTest2 for chloroplast phylogeny. The parameters for the AIC, which was used in maximum likelihood tree construction and BIC, which was used in MrBayes are given.

**Chloroplast phylogeny**							
**AIC**				**BIC**			
**Model**	TPM1uf+I+G			**Model**	TPM1uf+I		
**partition**	012210			**partition**	012210		
**-lnL**	213423.4102			**-lnL**	213427.9154		
**K**	53			**K**	52		
**freqA**	0.3090	**R(a)**	1.0000	**freqA**	0.3090	**R(a)**	1.0000
**freqC**	0.1906	**R(b)**	1.5668	**freqC**	0.1906	**R(b)**	1.5687
**freqG**	0.1916	**R(c)**	0.5249	**freqG**	0.1916	**R(c)**	0.5262
**freqT**	0.3088	**R(d)**	0.5249	**freqT**	0.3088	**R(d)**	0.5262
**ti/tv**	-	**R(e)**	1.5688	**ti/tv**	-	**R(e)**	1.5687
		**R(f)**	1.0000			**R(f)**	1.0000
**p-inv**	0.8800	**gamma**	2.3690	**p-inv**	0.9010	**gamma**	-

Supplemental Table S4: Parameters of the substitution model selected from the jModelTest2 for nuclear phylogeny. The parameters for the AIC, which was used in maximum likelihood tree construction and BIC, which was used in MrBayes are given.

**Nuclear phylogeny**							
**AIC**				**BIC**			
**Model**	GTR+I+G			**Model**	TIM3+I+G		
**partition**	012345			**partition**	012032		
**-lnL**	409805.0559			**-lnL**	409815.1532		
**K**	42			**K**	40		
**freqA**	0.2619	R(a)	1.1105	**freqA**	0.2614	**R(a)**	1.1946
**freqC**	0.2331	R(b)	3.5355	**freqC**	0.2331	**R(b)**	3.5323
**freqG**	0.2604	R(c)	1.0029	**freqG**	0.2609	**R(c)**	1.0000
**freqT**	0.2446	R(d)	1.2774	**freqT**	0.2446	**R(d)**	1.1946
**ti/tv**	-	R(e)	4.9048	**ti/tv**	-	**R(e)**	4.9019
		R(f)	1.0000			**R(f)**	1.0000
**p-inv**	0.2150	**gamma**	0.1930	**p-inv**	0.2160	**gamma**	0.1930

Supplemental Table S5: Details of the Indels inside CDS causing frameshifts and truncations of the proteins are given. The variants were determined compared to the reference *S. bicolor NC008602*.

**Taxon**	**Indels inside CDS**									
	**Polymorphism Type**	**Size (bp)**	**Sequence**	**Gene**	**Size of the gene (bp)**	**CDS position**	**Codon change**	**Amino acid change**	**Protein Effect**	**Conflict**
S. macrospermum 302367	Insertion	2	(T)3 -> (T)5	*rpl22*	438	436	no	no	Frame Shift	82342: Ref: T. Reads: A (4), C (2), G (1), T (5968)<br>82343: Ref: T. Reads: A (3), T (5971)<br>82344: Ref: T. Reads: A (1), G (1), T (5972)
S. macrospermum 326072	Insertion	2	(T)3 -> (T)5	*rpl22*	438	436	no	no	Frame Shift	82334: Ref T; Reads: -(15), G (3), T (2352)<br>82335: Ref T; -(15), G (1), T (2373)<br>82336: Ref T; A (2), T (2390)<br>82337: Ref T; A (3), T (2390)
S. laxiflorum 326060	Insertion	1	(T)10 -> (T)11	*rps11*	432	431	no	no	Frame Shift	77533^77534: Ref: -. Reads: - (2062), T (3)<br>77534: Ref: T. Reads: - (109), T (1868)<br>77535: Ref: T. Reads: - (202), T (1866)<br>77536: Ref: T. Reads: - (32), T (2033)<br>77537: Ref: T. Reads: - (25), T (2046)<br>77540: Ref: T. Reads: A (4), T (2066)<br>77541: Ref: T. Reads: G (1), T (2059)<br>77542: Ref: T. Reads: C (1), T (2053)
S. laxiflorum 326074	Insertion	1	(T)10 -> (T)11	*rps11*	432	431	no	no	Frame Shift	77533^77534: Ref: -. Reads: - (2062), T (3)<br>77534: Ref: T. Reads: - (204), T (1868)<br>77535: Ref: T. Reads: - (201), A (1), T (1866)<br>77536: Ref: T. Reads: - (32), T (2033)<br>77537: Ref: T. Reads: - (25), T (2046)<br>77540: Ref: T. Reads: A (1), T (2066)<br>77541: Ref: T. Reads: G (1), T (2059)<br>77542: Ref: T. Reads: C (1), T (2053)

S. leiocladum 326061	Insertion	21	TTTGTCTAAT GATTAATTA AG	*matK*	1548	1540	no	no	Truncation	1650: Ref: T. Reads: A (1), C (1), T (5785)<br>1651: Ref: T. Reads: A (2), G (2), T (5777)<br>1654: Ref: G. Reads: G (5452), T (4)<br>1655: Ref: T. Reads: A (1), C (1), T (5460)<br>1656: Ref: C. Reads: A (9), C (5457)<br>1657: Ref: T. Reads: G (1), T (5473)<br>1658: Ref: A. Reads: A (5446), C (1), G (2), T (1)<br>1659: Ref: A. Reads: A (5436), C (4), T (2)<br>1660: Ref: T. Reads: A (2), G (4), T (5447)<br>1661: Ref: G. Reads: G (5489), T (4)<br>1662: Ref: A. Reads: A (5493), C (1), T (1)<br>1663: Ref: T. Reads: A (3), T (5462)<br>1664: Ref: T. Reads: G (3), T (5474)<br>1665: Ref: A. Reads: A (5504), T (1)<br>1666: Ref: A. Reads: A (5489), C (2), T (4)<br>1667: Ref: T. Reads: A (1), G (3), T (5491)<br>1669: Ref: A. Reads: A (5462), G (1), T (2)<br>1670: Ref: A. Reads: A (5437), G (1), T (4)<br>1671: Ref: G. Reads: G (5441), T (9);
	Insertion	2	(T)10 -> (T)11	*rps11*	432	431	no	no	Frame Shift	77474: Ref: T, Reads: - (245), A (1), G (2), T (5951)<br>77474^77475: Ref: -. Reads: - (6179), C (1)<br>77475: Ref: T. Reads: - (69), A (1), T (6169)<br>77476: Ref: T. Reads: - (61), T (6183)<br>77477: Ref: T. Reads: G (1), T (6225)<br>77478: Ref: T. Reads: A (2), T (6222)<br>77479: Ref: T. Reads: A (2), T (6220)<br>77479: Ref: T. Reads: A (2), T (6220)<br>77479^77480: Ref: -. Reads: - (6091), A (1), C (1)<br>77480: Ref: T. Reads: A (2), C (1), G (2), T (6097)<br>77481: Ref: T. Reads: G (1), T (6206)<br>777483: Ref: T. Reads: A (1), C (1), G (5), T (6165)<br>7482: Ref: T. Reads: A (3), C (1), T (6168)<br>77482^77483: Ref: -. Reads: - (6155), G (1)<br>77483^77484: Ref: -. Reads: - (6142), G (1)<br>77484: Ref: T. Reads: G (2), T (6149)

S. leiocladum 326062	Insertion	21	TTTGTCTAAT GATTAATTA AG	*matK*	1548	1540	no	no	Truncation	1674: Ref: T. Reads: G (1), T (3603)<br>1675: Ref: G. Reads: G (3654), T (1)<br>1676: Ref: T. Reads: A (1), T (3649)<br>1677: Ref: C. Reads: A (2), C (3646)<br>1680: Ref: A. Reads: A (3626), C (2)<br>1681: Ref: T. Reads: C (5), G (1), T (3587)<br>1683: Ref: A. Reads: A (3647), G (1)<br>1684: Ref: T. Reads: A (1), G (6), T (3593)<br>1685: Ref: T. Reads: C (1), G (1), T (3603)<br>1686: Ref: A. Reads: A (3640), C (1)<br>1687: Ref: A. Reads: A (3665), T (2)<br>1688: Ref: T. Reads: A (3), G (1), T (3666)<br>1689: Ref: T. Reads: A (1), T (3666)<br>1690: Ref: A. Reads: A (3665), C (2)<br>1691: Ref: A. Reads: A (3619), G (1), T (2)
	Insertion	2	(T)10 -> (T)11	*rps11*	432	431	no	no	Frame Shift	77580: Ref: T. Reads: - (182), A (2), G (1), T (3760)<br>77581: Ref: T. Reads: - (58), A (1), T (3897)<br>77582: Ref: T. Reads: - (41), T (3918)<br>77583: Ref: T. Reads: - (7), T (3936)<br>77588: Ref: T. Reads: A (1), T (3911)<br>77588^77589: Ref: -. Reads: - (3905), A (1)<br>77589: Ref: T. Reads: A (1), C (1), G (1), T (3907)<br>77590: Ref: T. Reads: C (2), G (1), T (3893)<br>77591: Ref: A. Reads: A (3855), C (1)
S. matarankense 326065	Insertion	21	TTTGTCTAAT GATTAATTA AG	*matK*	1548	1540	no	no	Truncation	1671: Ref: G. Reads: G (3451), T (2)<br>1673: Ref: T. Reads: A (2), T (3429)<br>1674: Ref: T. Reads: C (1), T (3453)<br>1675: Ref: G. Reads: G (3509), T (1)<br>1677: Ref: C. Reads: A (3), C (3504)<br>1678: Ref: T. Reads: G (1), T (3503)<br>1679: Ref: A. Reads: A (3493), C (1), T (1)<br>1680: Ref: A. Reads: A (3485), C (2), T (1)<br>1681: Ref: T. Reads: A (1), G (1), T (3460)<br>1683: Ref: A. Reads: A (3499), T (3)<br>1684: Ref: T. Reads: C (1), G (5), T (3452)<br>1685: Ref: T. Reads: G (1), T (3454)<br>1686: Ref: A. Reads: A (3470), G (1)<br>1687: Ref: A. Reads: A (3489), C (2)<br>1688: Ref: T. Reads: G (1), T (3488)<br>1689: Ref: T. Reads: A (1), T (3485)<br>1691: Ref: A. Reads: A (3425), T (1)<br>1692: Ref: G. Reads: G (3693), T (1)

S. mataranksense 326065	Insertion	3	(T)3 -> (T)4	*rps11*	432	431	yes	yes	Insertion	77458: Ref: T. Reads: C (1), T (4170)<br>77459: Ref: T. Reads: G (2), T (4159)<br>77459^77460: Ref: -. Reads: - (4110), G (1)
	Insertion	5	TGGTT	*ycf2*	1987	1711	no	no	Frame Shift	86836: Ref T; Reads: -(5), A (2), C (1), T (3989)<br>86837: Ref G; -(5), A (2), G (4007)<br>86838: Ref G; -(5), A(1), T(4033)<br>86864: Ref T; -(5), A(1), T(4049)
S. mataranksense 326066	Insertion	21	TTTGTCTAAT GATTAATTA AG	*matK*	1548	1540	no	no	Truncation	1672: Ref: T. Reads: A (2), C (1), T (3460)<br>1673: Ref: T. Reads: C (1), G (3), T (3439)<br>1674: Ref: T. Reads: G (1), T (3475)<br>1675: Ref: G. Reads: G (3535), T (1)<br>1677: Ref: C. Reads: A (1), C (3524)<br>1678: Ref: T. Reads: C (1), G (1), T (3518)<br>1679: Ref: A. Reads: A (3502), C (2), G (1)<br>1680: Ref: A. Reads: A (3489), G (1), T (2)<br>1681: Ref: T. Reads: C (1), T (3465)<br>1682: Ref: G. Reads: G (3504), T (2)<br>1683: Ref: A. Reads: A (3495), G (1), T (2)<br>1684: Ref: T. Reads: A (3), G (2), T (3437)<br>1685: Ref: T. Reads: A (3), C (1), T (3440)<br>1686: Ref: A. Reads: A (3475), C (2)<br>1687: Ref: A. Reads: A (3486), T (1)<br>1688: Ref: T. Reads: G (2), T (3489)<br>1690: Ref: A. Reads: A (3484), T (1)<br>1691: Ref: A. Reads: A (3432), T (1)
	Insertion	3	(T)3 -> (T)4	*rps11*	432	431	yes	yes	Insertion	77341: Ref: T. Reads: A (2), T (3926)<br>77342: Ref: T. Reads: G (1), T (3922)<br>77343: Ref: T. Reads: C (1), G (1), T (3890)
	Insertion	5	TGGTT	*ycf2*	1987	1711	no	no	Frame Shift	86861: Ref T; Reads: -(4), A (1), C(4), T(4016)<br>86862: Ref G; -(4), A(1), G(4035)<br>86863: Ref G; -(4), A(1), T(4060)<br>86864: Ref T; -(4), C(1), G(1), T(4060)

S. purpureosericeum 326068	Insertion	21	TTTGTCTAATGATTAATTAAG	*matK*	1548	1540	no	no	Truncation	1672: Ref: T. Reads: A (2), G (1), T (6794)<br>1673: Ref: T. Reads: G (2), T (6757)<br>1675: Ref: G. Reads: G (6949), T (3)<br>1677: Ref: C. Reads: A (8), C (6922), G (17)<br>1679: Ref: A. Reads: A (6925), C (2)<br>1680: Ref: A. Reads: A (6908), C (3)<br>1681: Ref: T. Reads: G (2), T (6848)<br>1682: Ref: G. Reads: G (6936), T (6)<br>1683: Ref: A. Reads: A (6935), G (1), T (4)<br>1684: Ref: T. Reads: A (1), C (1), G (6), T (6830)<br>1685: Ref: T. Reads: A (1), C (1), G (2), T (6842)<br>1686: Ref: A. Reads: A (6921), C (1), G (1), T (2)<br>1687: Ref: A. Reads: A (6971), C (1), G (1)<br>1688: Ref: T. Reads: G (2), T (6979)<br>1689: Ref: T. Reads: A (1), C (1), T (6969)<br>1690: Ref: A. Reads: A (6970), T (1)<br>1691: Ref: A. Reads: A (6863), T (2)<br>1692: Ref: G. Reads: G (7363), T (7)

S. purpureosericeum 326068	Insertion	38	CACTGGGCG<br>GAATGGGAA<br>GACATAGTT<br>AGTTATTCTT<br>C	*rps3*	675	667	no	no	Truncation	81792 Ref: C. Reads: A (2), C (7470)<br>81794 Ref: C. Reads: A (3), C (7486)<br>81795 Ref: T. Reads: G (1), T (7110)<br>81796 Ref: G. Reads: G (7209), T (1)<br>81797 Ref: G. Reads: G (7167), T (9)<br>81798 Ref: G. Reads: A (1), G (7174), T (4)<br>81799 Ref: C. Reads: A (4), C (7175), T (1)<br>81800 Ref: G. Reads: A (1), G (7157), T (5)<br>81801 Ref: G. Reads: G (7305), T (1)<br>81802 Ref: A. Reads: A (7348), T (3)<br>81803 Ref: A. Reads: A (7392), C (13)<br>81804 Ref: T. Reads: G (1), T (7479)<br>81806 Ref: G. Reads: G (7413), T (1)<br>81807 Ref: G. Reads: A (9), G (7418), T (1)<br>81808 Ref: A. Reads: A (7449), T (1)<br>81809 Ref: A. Reads: A (7441), C (5)<br>81810 Ref: G. Reads: G (7488), T (1)<br>81811 Ref: A. Reads: A (7449), T (1)<br>81812 Ref: C. Reads: A (8), C (7475), G (1)<br>81814 Ref: T. Reads: - (9), G (4), T (7539)<br>81815 Ref: A. Reads: - (9), A (7505), C (1), T (1)<br>81816 Ref: G. Reads: - (9), G (7525), T (2)<br>81817 Ref: T. Reads: - (9), C (1), T (7529)<br>81818 Ref: T. Reads: C (1), G (1), T (7607)<br>81819 Ref: A. Reads: A (7408), T (2)<br>81820 Ref: G. Reads: G (7391), T (3)<br>81821 Ref: T. Reads: G (1), T (7400)<br>81822 Ref: T. Reads: A (2), C (1), T (7388)<br>81823 Ref: A. Reads: A (7346), C (3)<br>81824 Ref: T. Reads: A (2), G (1), T (7366)<br>81825 Ref: T. Reads: A (1), T (7342)<br>81826 Ref: C. Reads: A (1), C (7184)<br>81827 Ref: T. Reads: C (1), T (7096)<br>81828 Ref: T. Reads: A (2), C (14), T (7184)<br>81829 Ref: C. Reads: A (11), C (7183)
	Insertion	5	TGGTT	*ycf2*	1987	1711	no	no	Frame Shift	87496: Ref T; A(1), C(2), T(8140)<br>87497: Ref G; T(4), G(8164)<br>87498: Ref G; A(9), G(8234), T(4)<br>87499: Ref T; G(5), T(8248)
	Insertion	7	ATCCTAT	*ndhD*	1503	1500	no	no	Truncation	110882: Ref: A. Reads: A (7894), C (10), G (1)<br>110883: Ref: T. Reads: A (1), T (7898)<br>110884: Ref: C. Reads: A (7), C (7830)<br>110885: Ref: C. Reads: A (3), C (7835)<br>110887: Ref: A. Reads: A (7763), C (3)<br>110888: Ref: T. Reads: C (2), G (3), T (7759)

S. purpureosericeum 326071	Insertion	21	TTTGTCTAATGATTAATTAAG	*matK*	1548	1540	no	no	Truncation	1672: Ref: T. Reads: A (2), T (8661)<br>1673: Ref: T. Reads: G (3), T (8620)<br>1674: Ref: T. Reads: C (1), G (2), T (8696)<br>1675: Ref: G. Reads: A (2), G (8829), T (4)<br>1677: Ref: C. Reads: A (5), C (8757), G (25)<br>1677^1678: Ref: -------. Reads: ------- (8766), TAATGAT (1)<br>1678: Ref: T. Reads: A (2), C (2), G (1), T (8777)<br>1679: Ref: A. Reads: A (8749), T (1)<br>1680: Ref: A. Reads: A (8725), C (1), G (1), T (7)<br>1681: Ref: T. Reads: A (2), G (3), T (8646)<br>1682: Ref: G. Reads: C (1), G (8778), T (2)<br>1683: Ref: A. Reads: A (8766), C (1), T (3)<br>1684: Ref: T. Reads: A (2), G (4), T (8635)<br>1685: Ref: T. Reads: C (2), T (8642)<br>1686: Ref: A. Reads: A (8747), C (3)<br>1687: Ref: A. Reads: A (8780), C (1), G (2), T (1)<br>1688: Ref: T. Reads: G (3), T (8779)<br>1689: Ref: T. Reads: C (1), G (2), T (8775)<br>1690: Ref: A. Reads: A (8770), T (2)<br>1691: Ref: A. Reads: A (8624), T (4)<br>1692: Ref: G. Reads: A (1), G (9331), T (3)

S. purpureosericeum 326071	Insertion	38	CACTGGGCG GAATGGGAA GACATAGTT AGTTATTCTT C	*rps3*	675	667	no	no	Truncation	81566: Ref: C. Reads: A (5), C (9765), G (1), T (1)<br>81567: Ref: A. Reads: A (9787), T (3)<br>81568: Ref: C. Reads: A (3), C (9824)<br>81569: Ref: T. Reads: A (1), T (9283)<br>81570: Ref: G. Reads: G (9422), T (1)<br>81571: Ref: G. Reads: G (9366), T (9)<br>81572: Ref: G. Reads: G (9388), T (3)<br>81573: Ref: C. Reads: A (7), C (9386)<br>81574: Ref: G. Reads: C (1), G (9366), T (6)<br>81575: Ref: G. Reads: A (1), G (9600), T (6)<br>81576: Ref: A. Reads: A (9641), G (1), T (3)<br>81577: Ref: A. Reads: A (9735), C (10), T (2)<br>81578: Ref: T. Reads: A (1), T (9824)<br>81580: Ref: G. Reads: A (1), G (9747), T (5)<br>81581: Ref: G. Reads: A (7), G (9765), T (5)<br>81582: Ref: A. Reads: A (9806), C (1), G (1)<br>81583: Ref: A. Reads: A (9806), C (6), G (2)<br>81584: Ref: G. Reads: G (9820), T (4)<br>81585: Ref: A. Reads: A (9755), T (7)<br>81586: Ref: C. Reads: A (3), C (9790)<br>81587: Ref: A. Reads: A (9812), C (1), T (3)<br>81588: Ref: T. Reads: - (5), C (1), G (3), T (9867)<br>81589: Ref: A. Reads: - (5), A (9816), G (2), T (5)<br>81590: Ref: G. Reads: - (5), G (9854), T (3)<br>81591: Ref: T. Reads: - (5), A (5), C (1), G (1), T (9849)<br>81592: Ref: T. Reads: C (5), G (2), T (9933)<br>81593: Ref: A. Reads: A (9697), G (1), T (1)<br>81594: Ref: G. Reads: A (1), G (9692), T (1)<br>81595: Ref: T. Reads: C (2), G (1), T (9697)<br>81596: Ref: T. Reads: A (2), T (9692)<br>81597: Ref: A. Reads: A (9621), C (3), G (2), T (2)<br>81598: Ref: T. Reads: G (1), T (9634)<br>81599: Ref: T. Reads: G (2), T (9604)<br>81600: Ref: C. Reads: A (7), C (9410), G (1)<br>81601: Ref: T. Reads: A (2), C (1), G (3), T (9269)<br>81602: Ref: T. Reads: A (3), C (3), T (9421)<br>81603: Ref: C. Reads: A (10), C (9384), T (1)

S. purpureosericeum 326071	Insertion	5	TGGTT	*ycf2*	1987	1711	no	no	Frame Shift	87265: Ref T; Reads: -(9), A(1), C(2)<br>87268: Ref T; -(9), A(5), G(1), T(11026), G(10), T(10733)<br>87266: Ref G; -(9), A(16), G(10915), T(1)<br>87267: Ref G; -(9), G(10975)<br>87269: Ref T; -(9), A(1), C(1), G(5), T(10982)
	Insertion	7	ATCCTAT	*ndhD*	1503	1500	no	no	Truncation	110656: Ref: A. Reads: A (10973), C (15), G (1), T (2)<br>110657: Ref: T. Reads: A (2), C (1), T (10987)<br>110658: Ref: C. Reads: A (6), C (10902), G (1)<br>110659: Ref: C. Reads: A (11), C (10883), G (1)<br>110660: Ref: T. Reads: A (1), C (3), G (4), T (10618)<br>110661: Ref: A. Reads: A (10780), G (1), T (1)<br>110662: Ref: T. Reads: - (1), A (1), C (1), G (5), T (10765)
S. purpureosericeum 326075	Insertion	21	TTTGTCTAAT GATTAATTA AG	*matK*	1548	1540	no	no	Truncation	1672: Ref: T. Reads: A (1), C (2), G (1), T (8511)<br>1673: Ref: T. Reads: G (2), T (8458)<br>1674: Ref: T. Reads: A (3), G (2), T (8560)<br>1675: Ref: G. Reads: A (2), G (8792), T (5)<br>1676: Ref: T. Reads: A (2), C (1), T (8799)<br>1677: Ref: C. Reads: A (7), C (8750), G (34)<br>1678: Ref: T. Reads: C (3), G (2), T (8776)<br>1679: Ref: A. Reads: A (8752), C (1), G (1), T (1)<br>1680: Ref: A. Reads: A (8726), C (4), T (8)<br>1681: Ref: T. Reads: C (1), G (1), T (8662)<br>1682: Ref: G. Reads: C (1), G (8791), T (4)<br>1683: Ref: A. Reads: A (8781), G (1), T (3)<br>1684: Ref: T. Reads: A (1), G (10), T (8663)<br>1685: Ref: T. Reads: A (3), C (1), G (1), T (8681)<br>1686: Ref: A. Reads: A (8853), G (2)<br>1687: Ref: A. Reads: A (8901), C (1), G (1), T (1)<br>1688: Ref: T. Reads: A (1), G (1), T (8902)<br>1689: Ref: T. Reads: A (1), C (2), T (8892)<br>1690: Ref: A. Reads: A (8862), C (1), G (1), T (1)<br>1691: Ref: A. Reads: A (8724), T (6)<br>1692: Ref: G. Reads: G (9449), T (6)

S. purpureosericeum 326075	Insertion	38	CACTGGGCG GAATGGGAA GACATAGTT AGTTATTCTT C	*rps3*	675	667	no	no	Truncation	81627: Ref: C. Reads: A (5), C (8540), G (1)<br>81628: Ref: A. Reads: A (8558), G (1), T (5)<br>81629: Ref: C. Reads: A (7), C (8601)<br>81631: Ref: G. Reads: A (1), G (8275), T (2)<br>81632: Ref: G. Reads: A (2), G (8242), T (5)<br>81634: Ref: C. Reads: A (3), C (8243), T (1)<br>81635: Ref: G. Reads: A (3), G (8225), T (6)<br>81636: Ref: G. Reads: G (8415), T (1)<br>81637: Ref: A. Reads: A (8450), G (2), T (1)<br>81638: Ref: A. Reads: A (8516), C (12), G (2), T (1)<br>81639: Ref: T. Reads: G (2), T (8602)<br>81640: Ref: G. Reads: A (1), G (8487), T (3)<br>81641: Ref: G. Reads: G (8542), T (3)<br>81642: Ref: G. Reads: A (5), G (8538), T (2)<br>81643: Ref: A. Reads: A (8575), G (1), T (1)<br>81644: Ref: A. Reads: A (8576), C (5), G (2)<br>81645: Ref: G. Reads: A (1), G (8650)<br>81646: Ref: A. Reads: A (8616), G (1), T (1)<br>81647: Ref: C. Reads: A (4), C (8660)<br>81648: Ref: A. Reads: A (8674), C (2)<br>81649: Ref: T. Reads: - (4), A (1), C (2), G (2), T (8744)<br>81650: Ref: A. Reads: - (4), A (8715), G (3)<br>81651: Ref: G. Reads: - (4), G (8760)<br>81652: Ref: T. Reads: - (4), C (1), G (1), T (8768)<br>81654: Ref: A. Reads: A (8616), G (1), T (2)<br>81655: Ref: G. Reads: G (8598), T (3)<br>81656: Ref: T. Reads: A (3), T (8613)<br>81657: Ref: T. Reads: C (2), G (2), T (8604)<br>81659: Ref: T. Reads: G (1), T (8545)<br>81660: Ref: T. Reads: A (1), C (1), T (8519)<br>81661: : Ref: C. Reads: A (1), C (8335)<br>81662: Ref: T. Reads: A (1), C (1), G (1), T (8196)<br>81663: Ref: T. Reads: A (5), C (2), T (8322)<br>81664: Ref: C. Reads: A (9), C (8294)<br>81665: Ref: T. Reads: A (1), C (2), T (8340)
	Insertion	5	TGGTT	*ycf2*	1987	1711	no	no	Frame Shift	87331: Ref T; A(2), C(4), T(10336)<br>87332: Ref G; T(5), G(10336)<br>87333^87334: Ref -; -(4), -(10402), T(2)<br>87334: Ref T; A(1), C4), G(4), T(10421)

S. purpureosericeum 326075	Insertion	7	ATCCTAT	*ndhD*	1503	1500	no	no	Truncation	110717: Ref: A. Reads: A (9266), C (6)<br>110719: Ref: C. Reads: A (6), C (9186), T (1)<br>110720: Ref: C. Reads: A (4), C (9168)<br>110721: Ref: T. Reads: A (1), G (3), T (8945)<br>110722: Ref: A. Reads: A (9079), C (2), G (2)
S. brachypodum 302670	Insertion	21	TTTGTCTAAT GATTAATTA AG	*matK*	1548	1540	no	no		1672: Ref: T. Reads: - (1), T (3019)<br>1673: Ref: T. Reads: - (1), T (3002)<br>1674: Ref: T.Reads: - (1), T (3032)<br>1675: Ref: G. Reads: - (1), G (3073)<br>1676: Ref: T. Reads: - (1), T (3066)<br>1677: Ref: C. Reads: - (1), A (3), C (3055)<br>1678: Ref: T. Reads: - (1), C (1), G (1), T (3055)<br>1679: Ref: A. Reads: - (1), A (3033), C (3)<br>1680: Ref: A. Reads: - (1), A (3022), C (1), T (1)<br>1681: Ref: T. Reads: - (1), G (1), T (2993)<br>1682: Ref: G. Reads: - (1), G (3031)<br>1683: Ref: A. Reads: - (1), A (3028), G (1)<br>1684: Ref: T. Reads: - (1), G (3), T (2993)<br>1685: Ref: T. Reads: A (2), T (2999)<br>1686: Ref: A. Reads: A (3031), C (1)<br>1687: Ref: A. Reads: A (3039), C (1)<br>1690: Ref: A. Reads: A (3026), G (10)<br>1691: Ref: A. Reads: A (2990), G (1), T (1)<br>1692: Ref: G. Reads: G (3197), T (1)
	Insertion	2	(T)5 -> (T)6	*rps11*	432	431	no	no	Frame Shift	77444: Ref: T. Reads: - (203), T (2811)<br>77445: Ref: T. Reads: - (90), C (1), T (2954)<br>77446: Ref: T. Reads: - (49), T (3007)<br>77447: Ref: T. Reads: - (28), A (1), T (3023)<br>77448: Ref: T. Reads: - (8), C (1), T (3043)<br>77449: Ref: T. Reads: A (1), T (3055)<br>77454: Ref: T. Reads: C (2), G (1), T (3036)<br>77455: Ref: T. Reads: G (1), T (3029)
	Insertion	5	TGGTT	*ycf2*	1987	1711	no	no	Frame Shift	87029: Ref: T. Reads: C (1), G (2), T (3423)<br>87031: Ref: G. Reads: G (3453), T (1)<br>87033: Ref: T. Reads: A (1), G (1), T (3453)<br>87036: Ref: T. Reads: C (1), T (3625)

S. brachypodum 326073	Insertion	21	TTTGTCTAAT GATTAATTA AG	*matK*	1548	1540	no	no	Truncation	1672: Ref: T. Reads: A (1), G (2), T (3366)<br>1674: Ref: T. Reads: A (1), G (1), T (3388)<br>1675: Ref: G. Reads: G (3432), T (2)<br>1676: Ref: T. Reads: A (1), T (3430)<br>1677: Ref: C. Reads: A (3), C (3414), G (2)<br>1678: Ref: T. Reads: G (3), T (3413)<br>1679: Ref: A. Reads: A (3388), C (2), G (6), T (1)<br>1680: Ref: A. Reads: A (3378), C (2), T (4)<br>1681: Ref: T. Reads: A (1), G (1), T (3357)<br>1682: Ref: G. Reads: G (3405), T (1)<br>1683: Ref: A. Reads: A (3411), T (2)<br>1684: Ref: T. Reads: A (1), G (2), T (3360)<br>1685: Ref: T. Reads: A (1), T (3362)<br>1686: Ref: A. Reads: A (3394), G (1)<br>1687: Ref: A. Reads: A (3401), C (1), T (2)<br>1688: Ref: T. Reads: A (2), T (3405)<br>1689: Ref: T. Reads: A (2), T (3403)<br>1690: Ref: A. Reads: A (3405), T (1)<br>1692: Ref: G. Reads: C (1), G (3603), T (1)
	Insertion	4	(T)5 -> (T)7	*rps11*	432	431	no	no	Frame Shift	77460: Ref: T. Reads: - (251), A (1), T (3413)<br>77461: Ref: T. Reads: - (42), A (1), C (1), T (3668)<br>77462: Ref: T. Reads: - (3), C (1), T (3726)<br>77464: Ref: T. Reads: A (1), T (3748)<br>77465: Ref: T. Reads: A (1), T (3756)<br>77467: Ref: T. Reads: A (2), T (3706)<br>77468: Ref: T. Reads: A (1), C (1), G (1), T (3690)<br>77472: Ref: T. Reads: G (2), T (3745)<br>77473: Ref: T. Reads: A (1), T (3694)
	Insertion	5	TGGTT	*ycf2*	1987	1711	no	no	Frame Shift	87049: Ref: T. Reads: A (1), G (1), T (4386)<br>87050: Ref: T. Reads: A (2), T (4400)<br>87051: Ref: G. Reads: G (4410), T (4)<br>87051: Ref: G. Reads: G (4410), T (4)<br>87052: Ref: G. Reads: G (4435), T (1)<br>87053: Ref: T. Reads: C (2), T (4448)<br>87054: Ref: T. Reads: A (4), C (1), T (4447)
